# Single‐Nucleus and Spatial Transcriptome Profiling Delineates the Multicellular Ecosystem in Hepatocellular Carcinoma After Hepatic Arterial Infusion Chemotherapy

**DOI:** 10.1002/advs.202405749

**Published:** 2024-12-16

**Authors:** YeXing Huang, ZeFeng Du, ZhiCheng Lai, DongSheng Wen, LiChang Huang, MinKe He, ZiChao Wu, HuiFang Li, HanYue OuYang, WenChao Wu, Anna Kan, Ming Shi

**Affiliations:** ^1^ Department of Hepatobiliary Oncology Sun Yat‐sen University Cancer Center Guangdong Provincial Clinical Research Center for Cancer State Key Laboratory of Oncology in South China Guangzhou 510060 P. R. China

**Keywords:** hepatic arterial infusion chemotherapy, hepatocellular carcinoma, single‐cell Multi‐Omics, tertiary lymphoid structures, tumor immunology

## Abstract

Hepatic arterial infusion chemotherapy (HAIC) has emerged as a promising treatment strategy for hepatocellular carcinoma (HCC), but a detailed understanding of the multicellular ecosystem after HAIC treatment is lacking. Here, we collected tumor samples from treatment‐naïve primary and post‐HAIC HCC, and integrated single‐nucleus RNA sequencing with spatial transcriptomics to characterize the tumor ecosystem in the post‐HAIC HCC. Increased fractions and enhanced cellular communication of CD4^+^ T, CD20^+^ B, and dendritic cell subtypes were identified in post‐HAIC tumors. Moreover, it is substantiated that HAIC promoted tertiary lymphoid structures (TLS) formation, and addressed the roles of TLSs as spatial niches of cellular communication. Specifically, intermediate exhausted CD8^+^ T cells expressing Granzyme‐K and PD‐1 (PD‐1^+^CD8^+^ Tex‐int) expanded following HAIC and exhibited a functionally antitumor phenotype. PD‐1^+^CD8^+^ Tex‐int accumulated in the TLS vicinity and disseminated throughout the tumor microenvironment, demonstrating potential as an effective biomarker for HAIC‐based treatment in HCC. This study provides valuable resources and biological insights in the cellular underpinnings of HAIC treatment.

## Graphical abstract

By integrating single‐nucleus RNA sequencing, spatial transcriptomics, with multiplex immunohistochemistry, a spatially resolved atlas is provided for dynamics of immune and stromal cell compartment during Hepatic Arterial Infusion Chemotherapy (HAIC). This study provides new biological insights in the tumor ecosystem of post‐HAIC HCC, paving the road for the design of optimal HAIC‐immunotherapy combination strategies in future clinical practice.

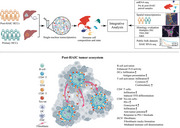

## 1 Introduction

Liver cancer, more specifically hepatocellular carcinoma (HCC), is the third leading cause of cancer‐related deaths and its incidence is increasing globally.^[^ ^1^ ^]^ Immunotherapy targeting the PD‐1/PD‐L1 axis has recently revolutionized the management of HCC.^[^ ^2^ ^]^ Moreover, our previous trials demonstrated that hepatic arterial infusion chemotherapy (HAIC) exhibited synergistic effects with PD‐1 blockade, and yielded significant anti‐tumor efficacy,^[^ ^3^, ^4^ ^]^ serving as one of the treatment options for patients with intermediate‐stage HCC.^[^ ^5^ ^]^ Despite these major advances, there is a lack of mechanistic research on the post‐HAIC tumor microenvironment.

Accumulating evidence indicates that the efficacy of chemotherapy not only involves direct cytotoxic effects but also relies on concomitant activation of antitumor immune responses.^[^ ^6^, ^7^ ^]^ However, the underlying mechanisms by which clinically effective HAIC drives cellular and microenvironmental components to elicit optimal immune responses and synergize with PD‐1 blockade are not completely understood. Cellular components within the tumor microenvironment (TME) play critical roles in the tumor biology and sensitivity to antitumor drugs.^[^ ^8^, ^9^ ^]^ Among the immune compartment, PD‐1^+^CD8^+^ T cells expressing intermediate level of checkpoint molecules and high level of effector genes have been associated with a potent response to immunotherapy in several studies.^[^ ^10^, ^11^ ^]^ Additionally, CD4^+^ T, B, and dendritic cells (DCs) have been reported to accumulate at intratumoral niches and induce antigen‐specific T‐cell responses,^[^ ^12^, ^13^, ^14^ ^]^ but how these cell types contribute to antitumor immunity after HAIC remains elusive.

Integrating single‐nucleus RNA sequencing (snRNA‐seq) with spatial transcriptomics (ST) has provided an avenue to explore the cellular components and their interactions in the TME at both cellular and spatial resolutions.^[^ ^15^ ^]^ Integrated analyses of the TME in solid tumors, such as nasopharyngeal carcinoma (NPC), colorectal cancer (CRC), and esophageal squamous cell carcinoma (ESCC) after immunotherapy or chemotherapy, have also been recently published,^[^ ^16^, ^17^, ^18^ ^]^ but it is not clear whether such findings can be applied to the study of HAIC treatment in HCC.

In this study, we conducted snRNA‐seq and ST on HCC specimens from treatment naive primary tumor (PT) and post‐HAIC tumor (HT) to systematically characterize the cellular and spatial heterogeneity of HCC after HAIC treatment. We demonstrated that HAIC promoted the formation of tertiary lymphoid structures (TLSs), and investigated the role of TLSs as spatial niches of cellular communication between CD4^+^ T, DC, and CD20^+^ B cells. Specifically, we identified a subset of intermediate exhausted CD8^+^ T cells with a functionally antitumor phenotype, which expanded after HAIC treatment, accumulated in the TLS vicinity, and disseminated along fibroblastic tracks, suggesting that this CD8^+^ T subset could be effective biomarker for HAIC therapy in patients with advanced HCC. Our integrated analysis delineated the tumor ecosystem in post‐HAIC HCC for the first time, and demonstrated the therapeutic potential of combining HAIC with PD‐1 blockade, which may guide the development of precision medicine to benefit a wide range of patients.

## 2 Results

### 2.1 Single‐Nucleus Transcriptome Atlas of Primary and Post‐HAIC HCC

**Figure 1:**
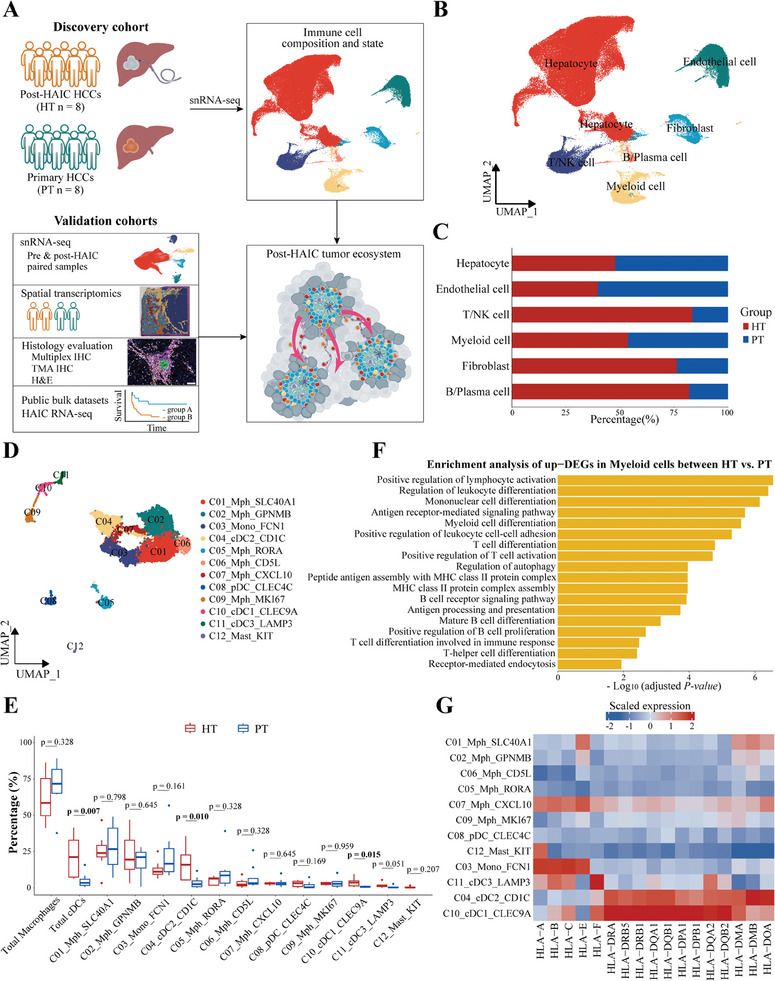
snRNA‐seq profiling of the tumor ecosystem in primary and post‐HAIC HCC. A) Schematic representation of the study design. B) Uniform Manifold Approximation and Projection (UMAP) plot showing the major cell types in the snRNA‐seq dataset. C) Bar plots indicating the fractions of major cell types originating from the HT or PT groups. D) UMAP plot showing the subtypes of myeloid cells. E) Boxplots illustrating the proportions of myeloid cell subtypes in HT and PT groups, respectively, and compared with a two‐sided Wilcoxon test (each group, n = 8). F) Bar chart showing GO pathway enrichment analysis of up‐regulated genes between the HT versus PT groups among myeloid cells. G) Heatmap indicating the expression of MHC‐I and MHC‐II molecules in the myeloid subtypes.

To characterize the cellular composition and dynamics during HAIC treatment, we prospectively collected 16 tumor samples of surgery and liver biopsies from 13 patients, and performed snRNA‐seq (Discovery cohort, **Figure** **1**A). Tumor samples from post‐HAIC HCC were referred to as HT group, whereas tumors from liver biopsy or primary HCC are referred to as PT group. To reduce heterogeneity and improve the reliability of our findings, we included six additional paired pre‐ and post‐treatment tumors, and conducted snRNA‐seq as Validation cohort 1. Moreover, we recruited six more patients with PT and HT samples for ST to create a spatial atlas (Validation cohort 2) as well as additional 20 patients for multiplex IHC (Validation cohort 3, Figure 1A). The detailed clinical and pathological characteristics are summarized in the Table S1 (Supporting Information).

In the discovery cohort, we obtained a high‐quality single‐cell transcriptome atlas for a total of 126 809 nuclei after removing low‐quality cells and doublets (Figure S1A and Table S2, Supporting Information). We annotated the cell clusters with canonical marker genes, which consisted of hepatocytes (*HNF4A*; *GPC3*), fibroblasts (*ACTA2*; *COL1A2*), endothelial cells (*CDH5*; *CD34*), T/NK cells (*CD2*; *IL7R*; *NKG7*), B/plasma cells (*BANK1*; *MS4A1*; *MZB1*) and myeloid cells (*CD86*; *CD163*; *LYZ*; Figure 1B; Figure S1B,C, Supporting Information). Although all these major cell types were shared across patients and treatments, the infiltration levels were apparently different (Figure S1D,E, Supporting Information). We found that HT group exhibited significant enrichment of T/NK, B/plasma cell and fibroblasts as compared to PT group (Figure 1C; Figure S1F,G, Supporting Information), possibly reflecting the substantial heterogeneity of ecosystem among primary and post‐HAIC HCC. These results were validated by performing snRNA‐seq in the 6 pre‐ and post‐treatment pairs of Validation cohort 1 (Figure S1H,I, Supporting Information), suggesting a microenvironmental remodeling effect of HAIC at the single‐cell level.

### 2.2 Characterization of the Heterogeneity of Intratumoral Dendritic Cells

We next performed unsupervised clustering of 7876 myeloid cells that were divided into 12 clusters, including macrophages, monocytes, DC, and mast cells (Figure 1D; Figure S2A,B, Supporting Information). We first analyzed the remodeling of myeloid cell subsets after HAIC therapy and found that only the fractions of conventional DCs (cDCs) significantly increased in HT group (total cDCs: *p* = 0.007; *C04_cDC2_CD1C: p* = 0.010; *C10_cDC1_CLEC9A*: *p* = 0.015; *C11_cDC3_LAMP3*: *p* = 0.051; Figure 1E). The snRNA‐seq profile for pre‐ and post‐treatment paired samples in the validation cohort 1 also revealed an increased abundance of cDCs in myeloid cells from post‐HAIC HCC samples (Figure S2C,D, Supporting Information). The primary function of cDCs in cancer immunity is to acquire tumor antigens, migrate to lymph nodes, and activate de novo T‐cell responses.^[^ ^19^ ^]^ In our discovery and validation cohorts, HAIC treatment was shown to increase the tumor infiltration of cDC subtypes. In addition, in post‐HAIC tumors, we also observed a significant increase in the antigen presentation signature levels, which suggested an enhancement of antigen presentation potential of myeloid cells (Figure S2E, Supporting Information). This result was further validated by functional enrichment analysis, which demonstrated that antigen processing and presentation, and antigen receptor mediated signaling pathway were enriched in myeloid cells from the post‐HAIC tumors. This result was established in both discovery and validation cohorts (Figure 1F; Figure S2F, Supporting Information). We therefore focused on the three cDC subtypes: cluster 4 highly expressed *CD1C*, *FCER1A*, *CLEC10A*, corresponding to cDC2; while cluster 10 highly expressed *CLEC9A*, *XCR1*, and *CADM1*, representing cDC1. Cluster 11 was reported to be “mature DCs”, due to increased expression of mature (*LAMP3*), migration (*CCR7*, *FSCN1*), and immunoregulatory molecules (*CD80*, *CD40*, *CCL22*, and *CD247*, Figure S2G, Supporting Information). As expected, *LAMP3* ^+^ DCs demonstrated the highest “activation and migration scores”, whereas cDC1 and cDC2 exhibited the powerful ability of antigen processing and presentation (Figure S2H, Supporting Information). Consistent with the findings of a prior study in NSCLC,^[^ ^12^ ^]^ cDCs exhibited increased expression levels of MHC‐II rather than MHC‐I molecules (Figure 1G), indicating that MHC‐II antigen presentation pathway was dominant after HAIC treatment. These findings prompted us to search for potential cDC‐CD4^+^ T cell interactions during HAIC treatment.

### 2.3 Distinct T Cell Compositions and States Between the Primary and Post‐HAIC HCC

**Figure 2:**
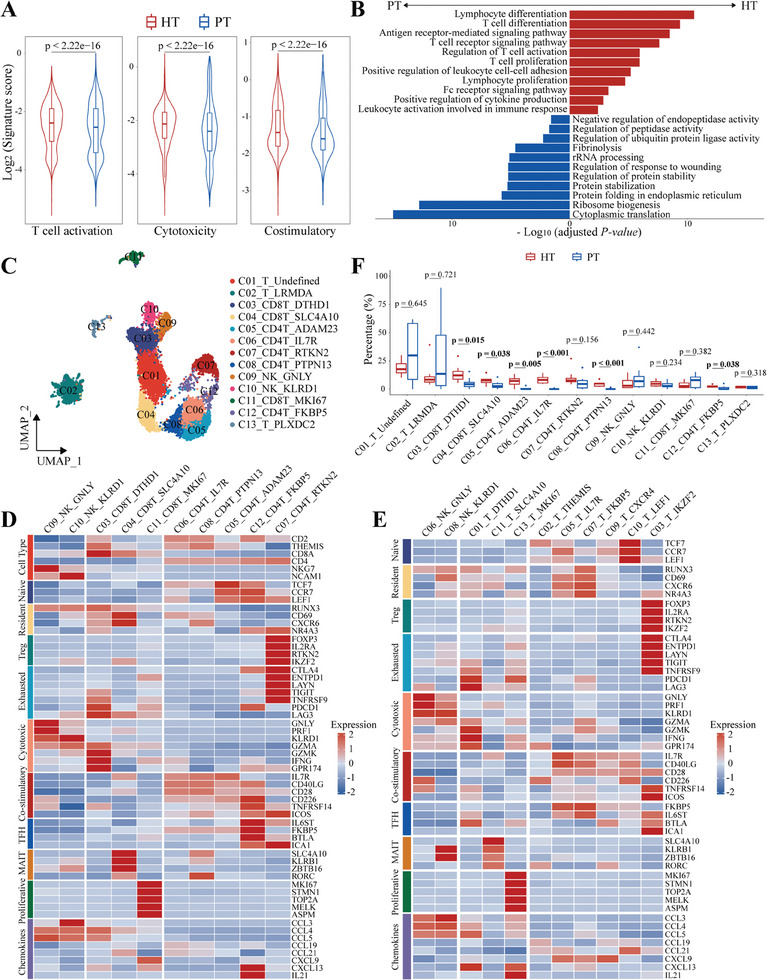
Distinct T cell compositions and states between primary and post‐HAIC HCC. A) Violin plots showing the expression scores of T cell activation, cytotoxicity, and co‐stimulatory signatures in T/NK cells from HT and PT samples, and compared with a two‐sided Wilcoxon test. B) Bar chart showing GO pathway enrichment analysis of differential expressed genes between the HT versus PT groups among T/NK clusters. C) UMAP plot showing the subtypes of T/NK cells. D) Heatmap indicating the expression of selected gene sets in the T/NK cell subtypes. E) Heatmap indicating the expression of selected gene sets in the T/NK cell subtypes from paired pre‐ and post‐HAIC samples in the validation cohort 1. F) Boxplots illustrating the proportions of T/NK cell subtypes in HT and PT groups, respectively, and compared with a two‐sided Wilcoxon test (each group, n = 8).

Since we observed remarkable divergence in T/NK cells between primary and post‐HAIC tumor ecosystem, we next investigated the unique transcriptional states of T cells following HAIC therapy. We first observed that T cells in post‐HAIC tumors presented increased expression of T cell activation, cytotoxicity, and co‐stimulatory signatures, which was also confirmed in the validation cohort 1 (**Figure 2**A; Figure S3A, Supporting Information). Differentially expressed gene (DEG) analysis revealed that the top up‐regulated genes included *CD69*, *IL7R*, *CXCR4*, and *CD28* (Figure S3B, Supporting Information), suggesting the activated and memory T cell phenotype in the post‐HAIC group. These results were confirmed with gene functional enrichment analysis in both discovery and validation cohorts, revealing that antitumor immunity programs were up‐regulated following HAIC therapy, and that the programs were composed of T cell proliferation and activation, T cell receptor signaling pathway, and antigen receptor‐mediated signaling pathway (Figure 2B; Figure S3C, Supporting Information).

To further identify the potential cell subtypes related to HAIC therapy, we performed sub‐clustering of 12323 T/NK cells, which led to the identification of 13 populations, each with its unique signatures (Figure 2C; Figure S3D,E, Supporting Information). Among CD4^+^ T cells, cluster 5 was identified as naïve T cells, highly expressing naïve markers (*TCF7*; *LEF1)*. Cluster 7 exhibited the highest expression levels of Treg markers (*FOXP3*, *IL2RA*, and *RTKN2*). *C12_CD4T_FKBP5* specifically showed features of chronic activation and exhaustion (*PDCD1*, *TOX2*, and *CTLA4*), and features of T follicular helper (T_FH_) cells^[^ ^20^ ^]^ including *BTLA*, *CXCL13*, and *FKBP5*. Similar analyses of CD8^+^ T cells revealed that cluster 4 displayed characteristics of mucosal‐associated invariant T cells (MAIT),^[^ ^21^ ^]^ including *SLC4A10*, *KLRB1*, and *ZBTB16* (Figure 2D). Although all these T subtypes were shared across patients and treatments, the infiltration levels were significantly different (Figure S3F, Supporting Information). SnRNA‐seq data of paired pre‐ and post‐HAIC tumors from validation cohort 1 also verified the similar cell clusters and expression patterns of marker genes, indicating that the definitions of cell clusters in our study were robust (Figure 2E; Figure S3G, Supporting Information).

Having identified candidate subtypes of T/NK cells that exhibited similarities to the previously described cytotoxic and exhausted populations, we further analyzed changes in frequencies of T/NK clusters to address whether tumor regression was driven mainly by T cells. We observed that HAIC therapy induced significant increases in the fractions of CD8^+^ T subsets (*C03_CD8T_DTHD1*: *p* = 0.015; *C04_CD8T_SLC4A10*: *p* = 0.038), naïve CD4^+^ T cells (*C05_CD4T_ADAM23*: *p* = 0.005), T_FH_ cells (*C12_CD4T_FKBP5*: *p* = 0.038), and T helper cells (*C06_CD4T_IL7R*, *C08_CD4T_PTPN13*: *p* < 0.001) in the HT samples (Figure 2F). The distribution of these subtypes suggested the important role of T cell program remodeling during HAIC treatment.

### 2.4 Enhanced Cellular Interactions Between CD4^+^ T, cDCs, and CD20^+^ B Cell Subtypes Contribute to Antitumor Immunity Following HAIC Treatment

**Figure 3:**
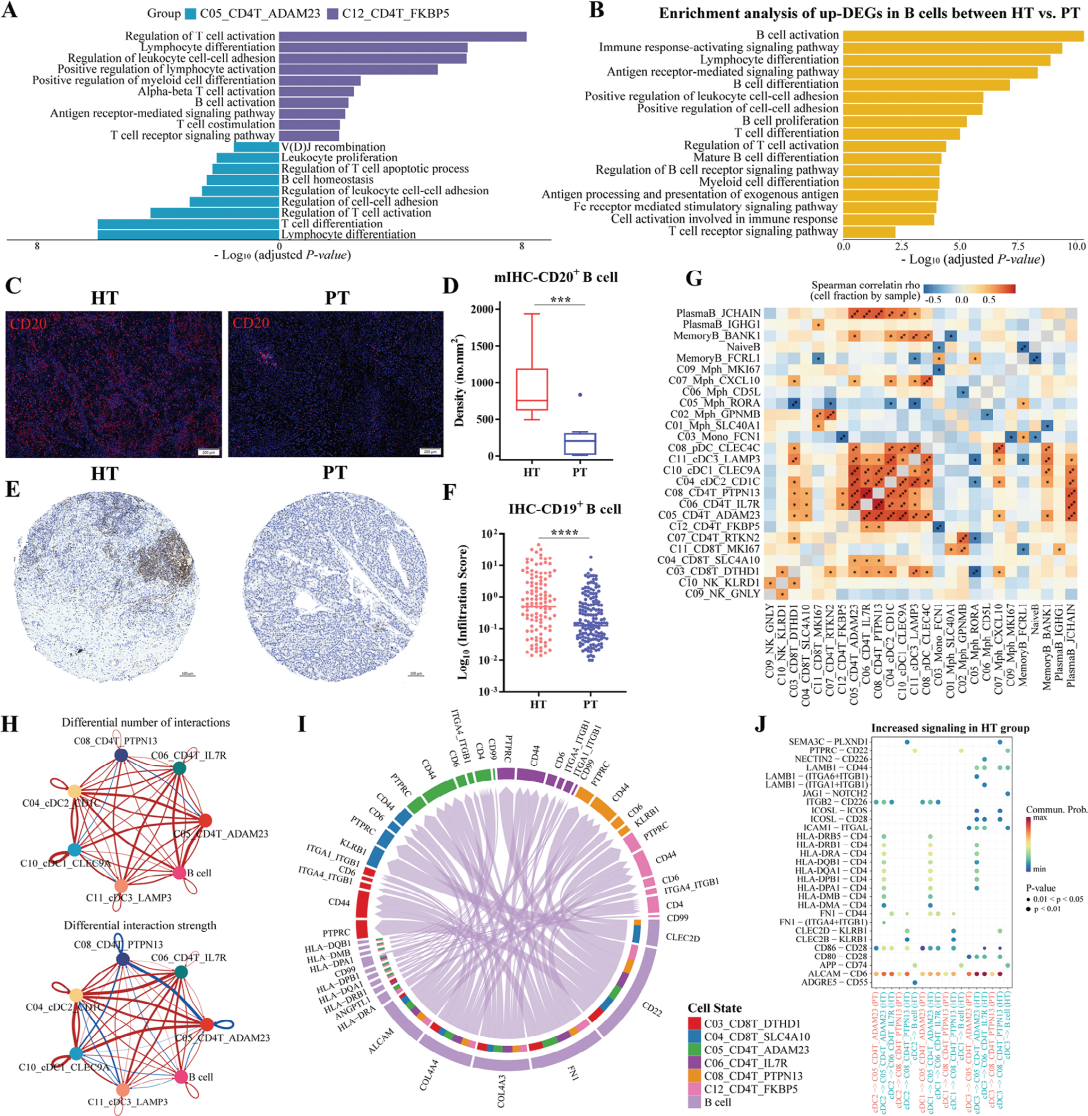
Enhanced cellular interactions between CD4^+^ T, cDCs, and CD20^+^ B cell subtypes contribute to antitumor immunity following HAIC treatment. A) Bar chart showing the differentially activated pathways in clusters *C05_CD4T_ADAM23* and *C12_CD4T_FKBP5*. B) Bar chart showing GO pathway enrichment analysis of up‐regulated genes between the HT versus PT groups among B cells. C) Representative images of multiplex IHC indicating CD20^+^ B cells in HT and PT samples. D) Boxplot showing the density of CD20^+^ B cells in each sample based on multiplex IHC staining images. Two‐sided Wilcoxon test, n = 20. E) Representative images of IHC staining on tissue microarrays indicating CD19^+^ B cells in HT and PT samples. F) Scatterplot showing the infiltration scores of CD19^+^ B cells in each sample according to IHC staining on tissue microarrays, and compared with a two‐sided Wilcoxon test. G) Heatmap showing the co‐occurrence pattern of immune cells. Spearman correlation analysis was performed according to cluster frequencies of immune cells in each sample. Color intensity is proportional to the Spearman correlation coefficient. H) Differences in the number and strength of cellular communications of various cell types between HT and PT groups. The blue line indicates decreased communications in the HT group, whereas the red line indicates increased communications in the HT group. The thicker the line, the greater the difference. I) Chord plot showing the receptor‐ligand analysis between B cells and T cell subtypes. J) Receptor‐ligand pairs that differ significantly between HT and PT groups based on cDC subtypes and other immune cell clusters. * *p* < 0.05, ** *p* < 0.01, *** *p* < 0.001, **** *p* < 0.0001.

As mentioned above, the fractions of naïve CD4^+^ T cells, T_FH_ cells, and T helper cells in the HT group were significantly increased (Figure 2F), indicating that HAIC therapy enhanced overall antitumor immunity by increasing CD4^+^ T cells. Moreover, T_FH_ cells in cluster *C12_CD4T_FKBP5* exhibited higher expression levels of *CXCL13* and *IL21* (Figure 2D), which were consistent with Magen et al.’s *CXCL13^+^IL21^+^PD‐1^+^CD4^+^* T helper cells^[^ ^14^ ^]^ and Li et al.’s *PD‐1^+^CXCR5^−^CD4^+^* Th‐*CXCL13* cell subsets,^[^ ^22^ ^]^ playing a prominent role in B cell chemotaxis and TLS formation. These findings were supported by the functional enrichment analyses of marker genes, suggesting that CD4^+^ T cells in cluster *C05_CD4T_ADAM23* and *C12_CD4T_FKBP5* might participate in lymphocyte differentiation and co‐stimulation, B cell activation and homeostasis (**Figure** **3**A).

Recent studies highlight the relevance of B cells and TLS in shaping immunotherapy response and promoting T cell‐mediated antitumor activity.^[^ ^23^, ^24^ ^]^ Through analyzing the transcriptome difference of B/Plasma cells after HAIC treatment, we first observed that B cells in post‐HAIC tumors exhibited a more activated functional state, due to greater enrichment in B cell proliferation, differentiation and activation, and immune response‐activating signaling pathways (Figure 3B). Moreover, the percentage of CD20^+^ B cells was significantly increased in the HT group, which was further validated by multiplex IHC (Figure 3C,D; Figure S4A,C, Supporting Information). Considering the relatively limited sample size of validation cohort 3, we performed immunohistochemical staining of tissue microarrays with HT and PT tumor samples and confirmed that the infiltration of B cells in tumor region was significantly increased in post‐HAIC samples (Figure 3E,F). Moreover, using the TLS hallmark genes,^[^ ^23^ ^]^ we observed a noteworthy increase in TLS activity following HAIC treatment (Figure S4D, Supporting Information).

To further explore the transcriptional similarity and co‐occurrence pattern of immune cell subtypes, we conducted Spearman correlation analysis of cell frequencies. Notably, we found that the abundances of CD4^+^ T, cDCs, and CD20^+^ B cell subsets were strongly positively correlated (Figure 3G). Moreover, the correlations of TLS activity with naïve CD4^+^ T (*C05_CD4T_ADAM23*) and T_FH_ cells (*C12_CD4T_FKBP5*) were validated by bulk RNA‐seq datasets (Figure S4E, Supporting Information), suggesting their co‐occurrence pattern in the TME. CellChat analysis exhibited the significantly increased number and strength of cellular communication between CD4^+^ T, cDCs and CD20^+^ B subtypes in the HT group (Figure 3H). Ligand receptor analysis showed that B cells displayed a strong potential to induce T cells into an activated phenotype via B‐T cell interactions (MHC‐II molecules/CD4, Figure 3I), addressing the role of B cells as antigen‐presenting cells (APCs) that initiate T‐cell antitumor immunity, especially in the post‐HAIC samples. Importantly, cell‐cell interaction analyses predicted that cDCs activated the functionality of CD4^+^ T and B cells through activated interactions (*CD86/CD80‐CD28*, *ICOSL‐ICOS*) to promote TLS formation and development (Figure 3J). The enhanced interactions between CD4^+^ T, cDCs, and CD20^+^ B cell subtypes may contribute to antitumor immunity by promoting TLS formation during HAIC treatment.

### 2.5 Spatial Co‐Localization of CD4^+^ T, cDCs, and CD20^+^ B Cells

**Figure 4:**
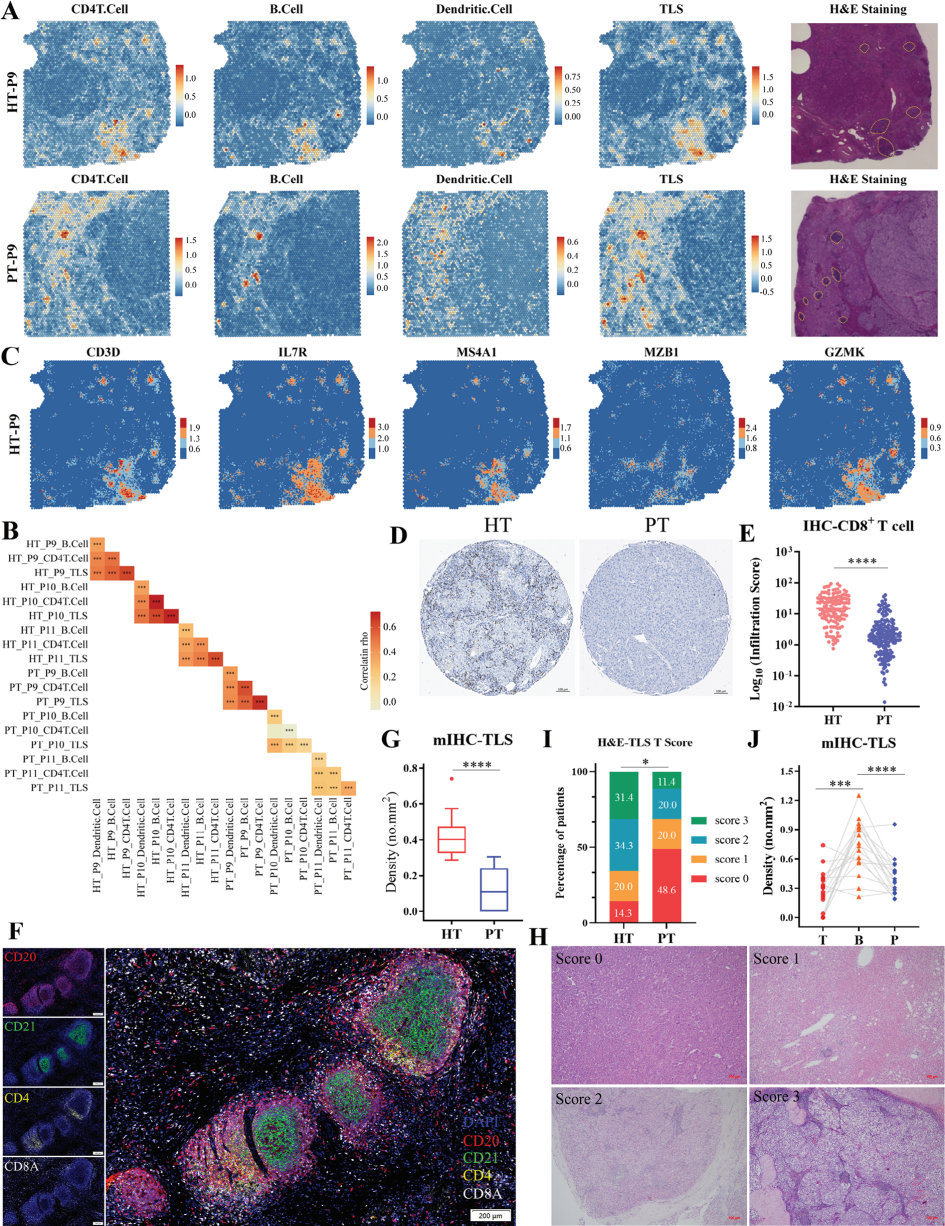
Spatial co‐localization of CD4^+^ T, cDCs, and CD20^+^ B cells. A) Spatial feature plots showing the signature scores of selected immune subtypes in spatial sections of 2 representative patients. Pathologist's annotations of the TLS areas on H&E staining used for the spatial transcriptomics assay were shown in right panel. B) Heatmap showing the Spearman correlation analysis on signature scores of selected immune subtypes in each sample of ST. Color intensity is proportional to the Spearman correlation coefficient. C) Spatial feature plots showing the enhanced expression of markers for T cells (*CD3D*, *IL7R*), B cells (*MS4A1*, *MZB1*), and cytotoxicity (*GZMK*) in one representative patient of HT group, calculated by BayesSpace algorithm. D) Representative images of IHC staining on tissue microarrays indicating CD8^+^ T cells in HT and PT samples. E) Scatterplot showing the infiltration scores of CD8^+^ T cells in each sample according to IHC staining on tissue microarrays, and compared with a two‐sided Wilcoxon test. F) Representative images of multiplex IHC indicating TLSs in the tumor region. G) Boxplot showing the density of intratumoral TLSs in each sample based on multiplex IHC staining images. Two‐sided Wilcoxon test, n = 20. H) Representative images of H&E staining indicating the TLS score systems in tumor core regions, I) Bar chart representing the percentages of patients with different TLS scores, n = 70. J) Comparison of the density of TLSs between paired tumor (T), boundary (B), and peri‐tumor (P) tissues in the multiplex IHC cohort, * *p* < 0.05, ** *p* < 0.01, *** *p* < 0.001, **** *p* < 0.0001.

Given the co‐occurrence patterns and cell‐cell interactions among CD4^+^ T, cDCs, and CD20^+^ B cells, we reasoned that these immune cells co‐localized to establish immune cell niches, such as TLSs. To investigate this possibility, we performed ST sequencing on tumor sections to acquire in situ gene expression profiles of six patients, with three PT and three HT tumor samples (Figure 1A). We first scored the spatial spots with gene signatures of CD4^+^ T, cDCs, and CD20^+^ B cells derived from the snRNA‐seq dataset, and highlighted the spatial co‐localization with these immune cells within the TME. By conducting H&E staining and TLS‐signature evaluation, we further confirmed that the spatial co‐localization of CD4^+^ T, cDCs, and CD20^+^ B cells was mainly concentrated at TLSs (**Figure 4**A; Figure S5A, Supporting Information). Moreover, the signature scores of TLS and specific immune subtypes exhibited the significantly positive correlations in spots of each patient in the ST dataset (Figure 4B), supporting their spatial co‐localization.

Increasing evidence suggests that cancer therapies, such as chemotherapy, radiotherapy, and immunotherapy, exert a profound effect on the formation and maturation of TLSs.^[^ ^25^ ^]^ Given the enhanced interactions and spatial co‐localization between CD4^+^ T, cDCs, and CD20^+^ B cells following HAIC therapy, we next sought to address whether HAIC could induce the formation of TLSs. BayeSpace‐enhanced spatial data revealed the abundant infiltration of T cells (*CD3D*, *IL7R*), B cells (*MS4A1*, *MZB1*), and cytotoxicity markers (*GZMK*) throughout tumor sections of HT samples, presenting an “immune‐activation” TME after HAIC therapy (Figure 4C; Figure S5B, Supporting Information). This “immune‐activation” phenotype was also observed in the tissue microarrays detected by immunohistochemical staining, which revealed increased infiltration scores of B and CD8^+^ T cells in the tumor regions of HT samples (Figures 3E,F and 4D,E).

To further investigate the ability of HAIC to induce TLS, we included an additional 20 patients for multiplex IHC and 70 patients for H&E staining. As shown in Figure 4F, CD20^+^ B cells were the major components of TLS, CD4 and CD8 staining indicated T cells enriched in the peripheral areas, and CD21 staining was used to detect follicular dendritic cells. As a result, we observed that HCC patients in the HT group exhibited a significantly higher density of TLS in tumor regions (Figure 4G), and this observation was confirmed by H&E staining. Among 35 the patients in the HT group, 5, 7, 12, and 11 patients were scored as 0, 1, 2, and 3, respectively, compared with 17, 7, 7, and 4 patients in the PT group (Figure 4H,I). Consistent with previous reports,^[^ ^26^ ^]^ we found TLSs were located primarily at the tumor margin (Figure 4J), providing more evidence to the premise that TLSs within tumor border were the front line of antitumor immune response. These results indicated a close relationship between TLS formation and HAIC treatment in HCC.

### 2.6 Intermediate‐Exhausted CD8^+^ T Subtype Increases Following HAIC Treatment and Exhibits Functionally Anti‐Tumor Phenotype

**Figure 5:**
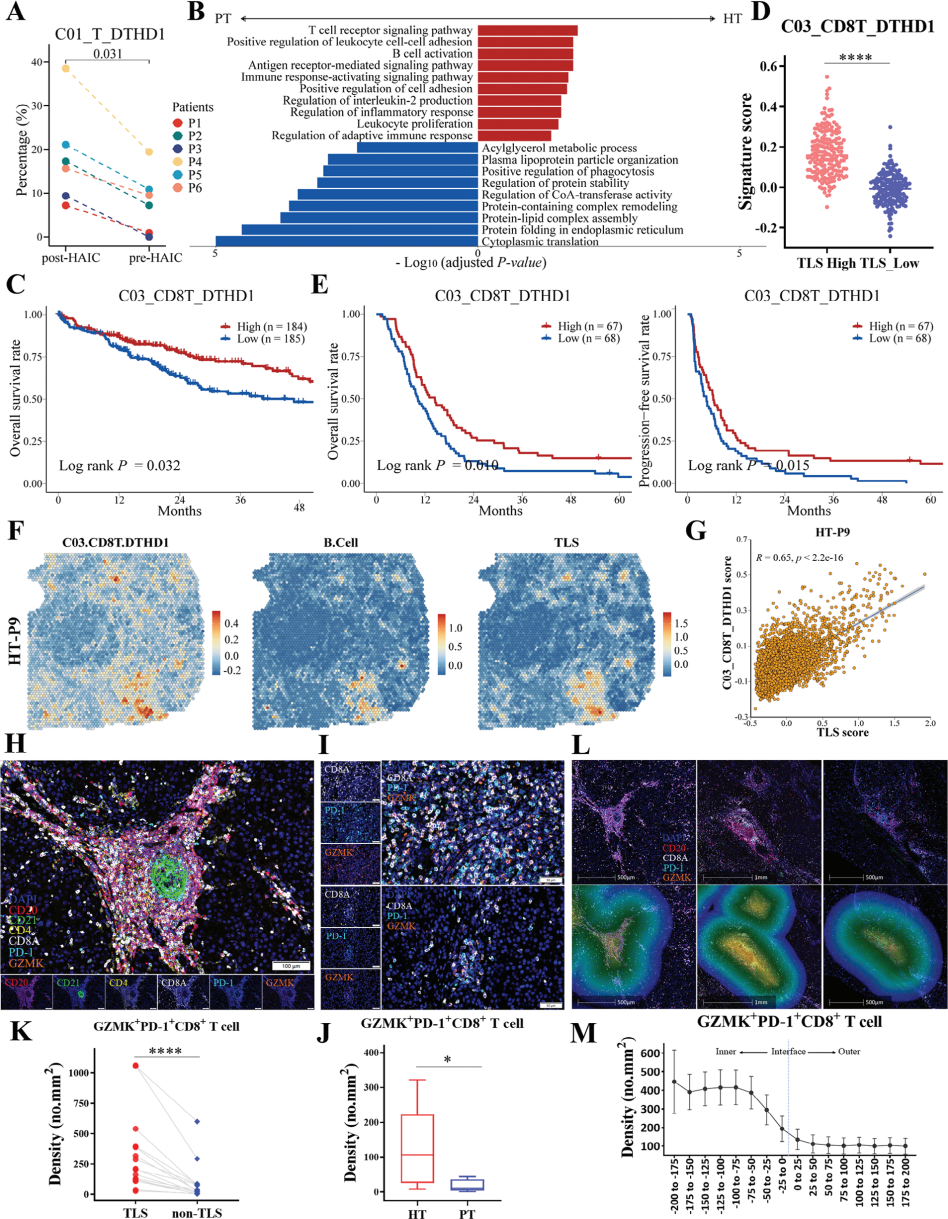
Intermediate‐exhausted CD8^+^ T subtype increases following HAIC and exhibits functionally antitumor phenotype. A) Comparison of percentage of cluster *C01_T_DTHD1* in the T/NK subtypes between paired pre‐ and post‐HAIC samples in the validation cohort 1, (each group, n = 6). B) Bar chart showing GO pathway enrichment analysis of differential expressed genes between the HT versus PT groups among cluster *C03_CD8T_DTHD1*. C) Kaplan‐Meier overall survival curves of TCGA‐LIHC cohort, grouped by the gene signature expression of cluster *C03_CD8T_DTHD1*. D) The scatterplot showing the comparison of *C03_CD8T_DTHD1* signature scores between the TLS‐high group and the TLS‐low group based on TCGA‐LIHC cohort. E) Kaplan‐Meier overall survival (OS) and progression‐free survival (PFS) curves of bulk RNA‐seq cohort with HCC patients receiving HAIC‐based treatment, grouped by the gene signature expression of cluster *C03_CD8T_DTHD1*. F) Spatial feature plots showing the signature scores of selected immune subtypes in spatial sections of one representative patient of HT group. G) Scatterplot showing the correlation analysis of signature score of *C03_CD8T_DTHD1* and TLS in spatial spots of one representative patient of HT group. H) Representative images of multiplex IHC indicating that Granzyme‐K^+^PD‐1^+^CD8^+^ T cells were primarily accumulated in TLSs. I) Representative images of multiplex IHC indicating Granzyme‐K^+^PD‐1^+^CD8^+^ T cells between the HT and PT groups. J) Boxplot showing the density of intratumoral GZMK^+^PD‐1^+^CD8^+^ T cells based on multiplex IHC staining images. Two‐sided Wilcoxon test, n = 20. K) Dotplot showing the comparison of density of intratumoral GZMK^+^PD‐1^+^CD8^+^ T cells located at the TLS vicinity versus paired non‐TLS regions, n = 16. L) Representative multiplex IHC staining images of infiltration bands of TLSs, and GZMK^+^PD‐1^+^CD8^+^ T cells distribution in three cases. M) The density of GZMK^+^PD‐1^+^CD8^+^ T cells within serial bands of TLSs. The dashed line indicates the interface line, n = 16, * *p* < 0.05, ** *p* < 0.01, *** *p* < 0.001, **** *p* < 0.0001.

Differences in cellular proportions of T cell subtypes between primary and post‐HAIC tumors were maximal for cluster *C03_CD8T_DTHD1* (Figure 2F). Consistent results from paired tumor samples were also obtained in the validation cohort 1, demonstrating that the percentage of *C01_T_DTHD1* was notably increased in post‐treatment samples (**Figure** **5**A). CD8^+^ T cells in *C03_CD8T_DTHD1* exhibited higher expression levels of cytotoxic markers (*GZMA*, *GZMK*, and *IFNG*) and moderately expressed exhaustion‐related molecules (*LAG3*, *TIGIT*, and *PDCD1*, Figure 2D,E), the combination of which was characteristic of intermediate‐exhausted CD8^+^ T cells (PD‐1^+^CD8^+^ Tex‐int).^[^ ^27^ ^]^ The trajectory analysis revealed that *C03_CD8T_DTHD1* cells represented an intermediate (transition) state between effector CD8^+^ and terminal‐exhausted CD8^+^ T cells^[^ ^21^ ^]^ (Figure S6A, Supporting Information). Following persistent exposure to tumor‐associated antigen, tumor‐specific CD8^+^ T cells displayed an exhausted state, which was characterized by up‐regulation of co‐inhibitory molecules and the progressive loss of proliferative capacity.^[^ ^28^, ^29^ ^]^ According to previous studies, tumor‐specific CD8^+^ T cells were largely composed by progenitor‐exhausted and terminal‐exhausted T cells.^[^ ^30^, ^31^ ^]^ Our analysis revealed similar results. We found that cluster *C03_CD8T_DTHD1* expressed high levels of characteristic markers of tumor‐specific CD8^+^ T cells, including *LAG3, PDCD1*, and *CXCL13* (Figure 2D‐E). In addition, cluster *C03_CD8T_DTHD1* exhibited greater cytotoxicity and exhaustion, but lower proliferation scores (Figure S6B, Supporting Information).

Previous evidence suggested that PD‐1^+^CD8^+^ Tex‐int cells were tumor antigen‐specific T cells and enhanced the response to PD‐1 blockade in NSCLC, ESCC, and HCC.^[^ ^14^, ^18^ ^]^ Therefore, we hypothesized that HAIC therapy synergized with PD‐1 blockade through increasing the tumor infiltration of PD‐1^+^CD8^+^ Tex‐int cells. In our discovery cohort, HAIC not only increased the proportions of *C03_CD8T_DTHD1*, but also up‐regulated pathways in T cell receptor signaling, immune response, and interleukin‐2 production (Figure 5B), suggesting a functionally antitumor phenotype after HAIC.

To investigate the clinical relevance of intermediate‐exhausted CD8^+^ T cells, survival analysis of TCGA‐LIHC and Fudan‐HCC databases revealed that *C03_CD8T_DTHD1* signature was associated with a better prognostic outcome in HCC patients (Figure 5C; Figure S6C, Supporting Information). Compared with tumors with a low abundance of TLSs, tumors with a higher abundance of TLSs exhibited higher *C03_CD8T_DTHD1* infiltration scores (Figure 5D; Figure S6D, Supporting Information). The findings supported the functionally antitumor phenotype of PD‐1^+^CD8^+^ Tex‐int cells in cluster *C03_CD8T_DTHD1*. To further investigate the predictive value of PD‐1^+^CD8^+^ Tex‐int cells in cluster *C03_CD8T_DTHD1* for HAIC‐based treatment in patients with HCC, we included an additional cohort with pre‐treatment tumor biopsy samples for bulk RNA‐seq (n = 135). Survival analysis revealed that a higher score for *C03_CD8T_DTHD1* signature was significantly associated with favorable progression‐free survival (PFS) and overall survival (OS) in patients with advanced HCC receiving HAIC‐based treatment (Figure 5E).

We next sought to address the spatial information of PD‐1^+^CD8^+^ Tex‐int cells in cluster *C03_CD8T_DTHD1*. ST sequencing data showed co‐localization of B cells with cluster *C03_CD8T_DTHD1*, particularly concentrated in TLSs (Figure 5F; Figure S6E, Supporting Information), which was validated by the positive correlation between TLS and *C03_CD8T_DTHD1* (Figure 5G; Figure S6F, Supporting Information). These spatial findings indicated that intermediate‐exhausted CD8^+^ T cells might accumulate at TLSs, which was consistent with the literature hypothesis that tumor‐reactive CD8^+^ T cell responses are present in TLSs.^[^ ^32^ ^]^ Additionally, PD‐1^+^CD8^+^ Tex‐int cells in cluster *C03_CD8T_DTHD1* are suggested to highly express Granzyme‐K but moderately express PD‐1. Multiplex IHC revealed that Granzyme‐K^+^PD‐1^+^CD8^+^ T cells were primarily accumulated in TLSs (Figure 5H), and the density of Granzyme‐K^+^PD‐1^+^CD8^+^ T cells in the tumor regions was significantly increased in the post‐HAIC samples (Figure 5H–J). A significantly increased level of Granzyme‐K^+^PD‐1^+^CD8^+^ T cells in the neighboring regions of TLSs was also detected (Figure 5K). Spatially, we characterized the density of Granzyme‐K^+^PD‐1^+^CD8^+^ T cells within 200µm of the inner and outer edges of TLSs and their distance to the interface (Figure 5L). As a result, we observed that Granzyme‐K^+^PD‐1^+^CD8^+^ T cells were predominantly located in the TLS vicinity, and the density decreased significantly with increasing distance to the interface (Figure 5M).

Collectively, these results confirmed that HAIC therapy increased the accumulation of intermediate‐exhausted Granzyme‐K^+^PD‐1^+^CD8^+^ T cells with a functionally antitumor phenotype, which predominantly located in the TLS vicinity.

### 2.7 Intermediate‐Exhausted CD8^+^ T Cells Accumulate in TLSs and Disseminate Along Fibroblastic Tracks

**Figure 6:**
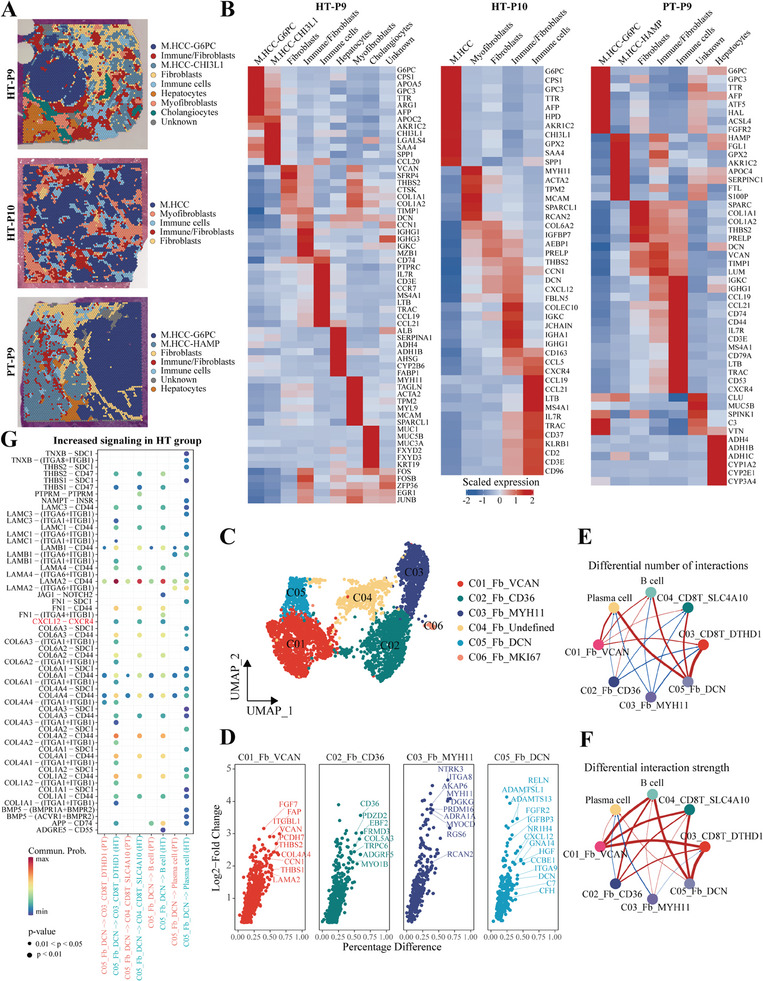
Intermediate‐exhausted CD8^+^ T subtypes accumulate in TLSs and disseminate along fibroblastic tracks. A) Unbiased clustering of ST spots and definition of cell types of each cluster in tumor sections of three representative patients. B) Heatmap showing the average expression of known markers in indicated clusters. C) UMAP plot showing the subtypes of fibroblasts. D) scVolcano plots showing the marker genes in each cluster of fibroblasts. E, F) Differences in the number (E) and strength (F) of cellular communications from fibroblasts to immune cell types between HT and PT groups. G) Receptor‐ligand pairs that differ significantly between HT and PT groups based on fibroblasts and other immune cell clusters.

As shown in Figure 5, PD‐1^+^CD8^+^ Tex‐int cells in cluster *C03_CD8T_DTHD1* were spatially distributed in the TLS vicinity and gradually decreased at distance, indicating that CD8^+^ T cells disseminated from TLSs into the tumor bed. To prove this possibility, we first conducted an unbiased clustering of the ST dataset, and the tumor tissues were further classified as hepatocytes, myofibroblasts, fibroblasts, cholangiocytes, malignant hepatocytes, immune cells, and endothelial cells (**Figure** **6**A,B; Figure S7A,B, Supporting Information). Intriguingly, we identified a specially spatial cluster that simultaneously highly expressed signatures of immune cells and fibroblasts, which was designated as immune/fibroblasts (Figure 6A,B; Figure S7A,B, Supporting Information). The two cell types could not be distinguished in this cluster, as they were co‐localized in the same ST spots, suggesting the physical interaction and spatial crosstalk between these cell types.^[^ ^33^ ^]^ These results suggested that fibroblasts might be involved in mediating the migration of immune cells, including intermediate‐exhausted CD8^+^ T cells in *C03_CD8T_DTHD1*.

To identify the potential fibroblast subtypes involved in T cell migration, we next sub‐clustered fibroblast compartment in the snRNA‐seq data (Figure 6C; Figure S7C, Supporting Information). Among them, *C05_Fb_DCN* expressed highest levels of *CXCL12*, *DCN*, and *IGFBP3* (Figure 6D), which was consistent with Qin et al.’s remodeled CAF subtypes and was strongly associated with chemotherapy response.^[^ ^34^ ^]^ The remodeled CAF subtypes were reported to regulate the TME through spatial recruitment and crosstalk to activate immunity, and suppress tumor progression through multiple cytokines, including *CXCL12*, and *DCN*.^[^ ^34^ ^]^ Functional enrichment analysis indicated that DCN^+^ fibroblasts in *C05_Fb_DCN* might participate in extracellular structure organization, cell‐cell junctions, and immune cell chemotaxis (Figure S7D, Supporting Information). Furthermore, ST data showed that cluster immune/fibroblasts expressed high levels of *DCN* (Figure 6B; Figure S7B, Supporting Information), therefore, we supposed that DCN^+^ fibroblast subsets might be involved in immune cell migration, including CD8^+^ T cells in *C03_CD8T_DTHD1*. Interestingly, CellChat analysis showed the increased number and strength of cell‐cell interactions between *C05_Fb_DCN* and *C03_CD8T_DTHD1* after HAIC treatment (Figure 6E,F), which were concentrated on collagen, laminin, and integrin pathways, supporting their directly physical interaction (Figure 6G). Moreover, this interaction was confirmed by showing the significant positive correlation between *C05_Fb_DCN* and *C03_CD8T_DTHD1* in bulk RNA‐seq datasets (Figure S7E, Supporting Information).

**Figure 7:**
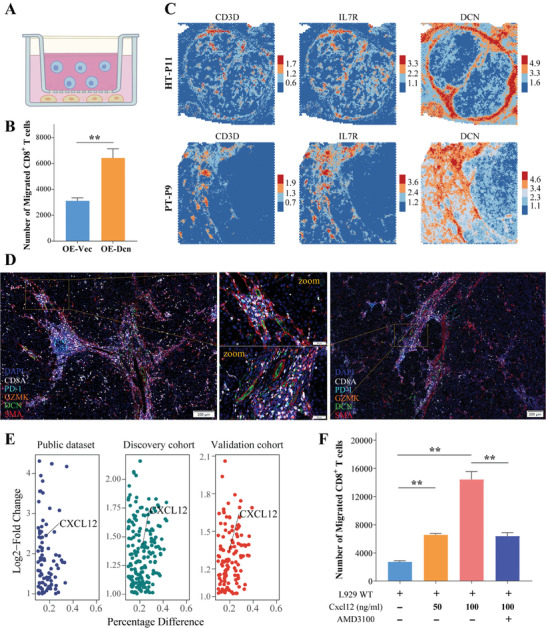
DCN^+^ fibroblasts may participate in lymphocyte recruitment and dissemination. A) Schematic representation of the transwell chemotaxis assay. B) Boxplot showing the quantification of migrated CD8^+^ T cells from in vitro transwell chemotaxis assay, (each group, n = 6). C) Spatial feature plots showing the enhanced expression of markers for T cells (*CD3D*, *IL7R*) and *DCN* in two representative patients, calculated by BayesSpace algorithm. D) Representative images of multiplex IHC indicating that Granzyme‐K^+^PD‐1^+^CD8^+^ T cells were located at a very close proximity to DCN^+^ fibroblasts. E) ScVolcano plots derived from integrated analysis, discovery, and validation cohorts confirmed that DCN^+^ fibroblasts exhibited higher expression level of *CXCL12*, when compared with DCN^−^ fibroblasts. F) Boxplot showing the quantification of migrated CD8^+^ T cells from in vitro transwell chemotaxis assay in the presence or absence of *Cxcl12*, (each group, n = 6). * *p* < 0.05, ** *p* < 0.01, *** *p* < 0.001, **** *p* < 0.0001.

To further investigate the chemotaxis of DCN^+^ fibroblasts, we generated a stable *Dcn* overexpression mouse fibroblast cell line (L929, Figure S7F,G, Supporting Information), and established an in vitro transwell migration dual‐chamber model (**Figure** **7**A). From the chemotaxis assay, we found a significantly increased number of migrated CD8^+^ T cells, when co‐cultured with Dcn^+^ fibroblasts (Figure 7B). Spatially, we observed a similar distribution pattern of T cell markers (*CD3D*, *IL7R*) with *DCN*, suggesting the co‐localization of T cells and DCN^+^ fibroblasts (Figure 7C). Moreover, multiplex IHC demonstrated that DCN^+^ *SMA^+^* fibroblasts assembled into a dense network, which was designated as fibroblastic track (Figure 7D). We noticed that intermediate‐exhausted Granzyme‐K^+^PD‐1^+^CD8^+^ T cells are mainly located at a very close proximity to DCN^+^ fibroblastic tracks (Figure 7D). These findings raised a possibility that DCN^+^ fibroblasts exhibited chemotaxis to CD8^+^ T cells, potentially mediating the spatial recruitment, interaction, and dissemination of CD8^+^ T cells within the TME.

We next investigated how DCN^+^ fibroblasts modulated CD8^+^ T cells. Ligand receptor analysis revealed that cellular interactions between *CXCL12* expressed by fibroblasts and *CXCR4* expressed by CD8^+^ T cells were enriched specifically in post‐HAIC tumors (Figure 6G). The sc/snRNA‐seq profile of multiple datasets also confirmed that DCN^+^ fibroblasts exhibited significantly higher expression of *CXCL12*, compared with DCN^−^ fibroblasts (Figure 7E). Furthermore, transwell chemotaxis assay revealed that *Cxcl12* induced the migration of CD8^+^ T cells in a dose‐dependent manner, whereas the *CXCR4* inhibitor AMD3100 partially blocked the migration of CD8^+^ T cells (Figure 7F). Taken together, these results suggested that DCN^+^ fibroblasts might participate in lymphocyte recruitment partially via the *CXC12‐CXCR4* axis, particularly in post‐HAIC tumors.

### 2.8 Intratumoral Transcriptomic Heterogeneity of Malignant Hepatocytes

**Figure 8:**
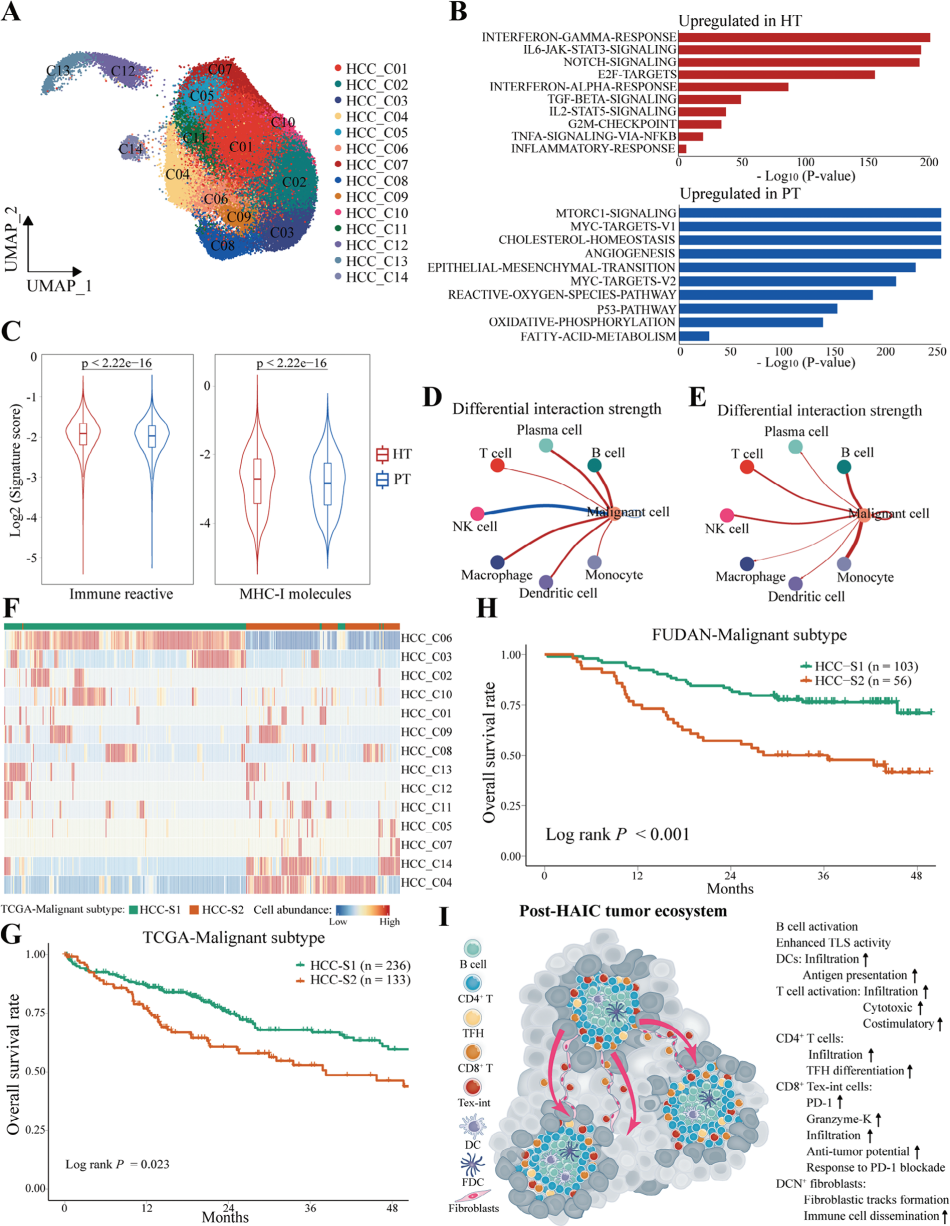
Characterization of the transcriptomic heterogeneity of intratumoral malignant hepatocytes. A) UMAP plot showing the subtypes of malignant hepatocytes. B) Bar charts showing the enrichment of specific pathways, according to the hallmark gene sets in malignant hepatocytes from HT and PT groups. C) Violin plots showing the signature scores of immune reactive and MHC‐I molecules in malignant hepatocytes from HT and PT samples, and compared with a two‐sided Wilcoxon test. D) Differences in the strength of cellular communications from immune subtypes to malignant hepatocytes between HT and PT groups. E) Differences in the strength of cellular communications between immune cells and malignant cells from paired pre‐ and post‐HAIC samples in the validation cohort 1. F) The heatmap showing two malignant subtypes of HCC patients, with distinct abundance of malignant cell subtypes inferred from TCGA‐LIHC cohort. G) Kaplan‐Meier overall survival curves of TCGA‐LIHC cohorts, grouped by the two malignant subtypes. H) Kaplan‐Meier overall survival curves of Fudan‐HCC cohorts, grouped by the two malignant subtypes. I) Schematic diagram of the unique tumor ecosystem in post‐HAIC HCC.

Our analysis revealed substantial differences in the immune and stromal compartments of tumor ecosystem between primary and post‐HAIC HCC, which led us to investigate their profound effects on malignant cells. By calculating the copy number variation with CopyKAT, we identified a total of 61779 malignant cells that were separated into 12 clusters, enabling us to systemically explore their intratumoral heterogeneity (**Figure 8**A; Figure S8A, Supporting Information). We first found that HT samples expressed higher scores of proliferative signature, whereas PT samples presented higher signals of hepatic and cancer stemness (Figure S8B, Supporting Information). Additionally, we observed an enrichment of immune‐related pathways in HT group (e.g., INF‐gamma/alpha‐signaling, IL6‐JAK‐STAT3‐signaling, IL2‐STAT5‐signaling, and TNFA‐signaling‐via‐NFKB, Figure 8B), suggesting that malignant cells in the HT group exhibited an inflammatory state, which might drive more interactions with the immune system after HAIC treatment. In line with this inflammatory state, the signatures of immune reactive and MHC‐I pathways were significantly up‐regulated in malignant cells from post‐HAIC tumors (Figure 8C), which were confirmed in snRNA‐seq data of paired samples from validation cohort 1 (Figure S8C,D, Supporting Information). The importance of these inflamed malignant hepatocytes was emphasized by cell‐cell interaction analysis, exhibiting strong interactions between malignant and immune cell subtypes in the post‐HAIC HCC in both discovery and validation cohorts (Figure 8D,E). In summary, these results disclose HAIC modulating the inflammatory program of malignant cells.

Next, we sought to identify malignant subtypes of HCC based on cell abundances of malignant cell subsets inferred from TCGA‐LIHC and Fudan‐HCC cohorts. Consensus clustering identified two distinct cellular modules (HCC‐S1 and HCC‐S2), which were associated with significantly different clinical outcomes (Figure S8E, Supporting Information). Tumor in HCC‐S1 subtype exhibited higher proportions of HCC‐C06 and HCC‐C03 subclusters, HCC‐S2 contained higher fractions of cluster HCC‐C04 and HCC‐C14 (Figure 8F; Figure S8F, Supporting Information). These malignant subtypes were verified in the Fudan‐HCC cohort, revealing that HCC‐S1 subtype was significantly associated with favorable OS in patients with HCC (Figure 8G,H). Progress in the last decade has revealed that cancer is a metabolic disorder in which several metabolic pathways are rewired to enhance cell proliferation.^[^ ^35^ ^]^ We next quantified the metabolic activity of single cells, and found that HCC‐C06 and HCC‐C03 exhibited the higher metabolic activities (Figure S8G, Supporting Information). To further investigate the Hallmark landscape of these malignant subtypes and determine their association with previously reported molecular subclasses of HCC. Consistent with subgroup S‐Mb in Gao's study,^[^ ^36^ ^]^ HCC‐S1 subtype was characterized by enrichment of pathways related to biological metabolism, and was thus designated as metabolism‐dominated phenotype. While HCC‐S2 exhibited higher enrichment of cell‐cycle‐related pathways, showing common similarity with S‐Pf subgroup, and was therefore designated as proliferation‐dominated subtype (Figure S8H, Supporting Information).

## 3 Discussion

FOLFOX‐HAIC has emerged as a promising treatment strategy for advanced HCC. Adaptive immune response activation is critical for long‐term tumor control by the local chemotherapy; however, the mechanism by which HAIC triggers immune response and synergizes with PD‐1 blockade is poorly understood. By employing a comprehensive multi‐omics approach, this study provided the most comprehensive single‐cell profiling of the ecosystem in post‐HAIC HCC, not only presenting a high‐resolution description of cellular diversity at the spatial and temporal levels, but also delineating the molecular underpinnings of combining HAIC with PD‐1 blockade.

In this study, we revealed a distinct immune ecosystem in post‐HAIC HCC (Figure 8I), characterized by the increased proportions of cDCs, CD4^+^ T, B cell subtypes, and intermediate‐exhausted Granzyme‐K^+^PD‐1^+^CD8^+^ T cells. Notably, we observed that T and B cells exhibited an activated functional state, and DCs enhanced the efficiency of antigen presentation during HAIC treatment, indicating the immunomodulatory effects of HAIC. As a result, malignant cells in post‐HAIC tumors exhibited an inflammatory state and up‐regulated expression of MHC‐I molecules for a direct modulation of tumor immunogenicity. These results suggested that clinically effective HAIC induced active crosstalk between malignant hepatocytes and immune cells in HCC, which may be one of the mechanisms for synergizing with PD‐1 blockade. The observation was evidenced by the recent success of HAIC‐immunotherapy combined strategies in clinical practice.^[^ ^4^, ^37^ ^]^

Our analyses demonstrated that the co‐occurrence pattern and cellular interactions of CD4^+^ T, cDCs, and CD20^+^ B cell subtypes occurred in TLSs. TLSs are characterized by organized aggregates of CD20^+^ B and CD3^+^ T cells, associated with superior prognosis and response to immunotherapy.^[^ ^25^ ^]^ Within the T cell compartment, T_FH_ cells represent the dominant subsets, but CD8^+^ cytotoxic T and CD4^+^ T helper cells are also present.^[^ ^38^ ^]^ In this study, we observed that HAIC treatment increased the proportions of naïve CD4^+^ T, T_FH_, and T helper cells. T_FH_ cells in *C12_CD4T_FKBP5* highly expressed *CXCL13* and *IL21*. *CXCL13* acted specifically to recruit and position *CXCR5^+^* cells in lymphoid follicles, primarily *CXCR5^+^* B cells.^[^ ^39^ ^]^ Overexpression of *CXCL13* in an inflamed tissue appeared to promote the formation of functional TLSs, and activate humoral anti‐tumor response.^[^ ^40^ ^]^ *IL‐21* produced by T_FH_ cells played an important role in B cell proliferation, activation, and differentiation.^[^ ^41^ ^]^ HAIC treatment exhibited the ability to reshape B cell plasticity and promote TLS formation, as evidenced by the increased density of CD20^+^ B cells and TLSs in post‐HAIC tumors. B cells exhibited strong regulatory potential to initiate T cell antitumor immunity via B‐T interactions, especially in post‐HAIC tumors. A possible explanation was that HAIC‐induced release of tumor antigens was processed and presented by cDCs and B cells to elicit antigen‐specific T cell response, promoting naïve CD4^+^ T cells differentiation into T_FH_ cells and T helper cells. The HAIC‐induced remodulation to a more immunogenic TME may be another mechanism for synergizing with immunotherapy.

The diversity of PD‐1^+^CD8^+^ T cells have previously been well described, which were associated with better response to immunotherapy in several clinical studies.^[^ ^10^, ^11^ ^]^ Here, we identified a distinct CD8^+^ T cell composition enriched in post‐HAIC tumors, which were intermediate‐exhausted Granzyme‐K^+^PD‐1^+^CD8^+^ T cells with potent antitumor activity, representing a tumor‐reactive phenotype.^[^ ^28^ ^]^ Multiple reports have characterized the phenotypes of tumor‐reactive CD8^+^ T cells, and suggested that exhaustion‐related genes (*PD‐1*, *CXCL13*, *LAG3*) are capable of discriminating tumor‐reactive from virus‐specific bystander clones within treatment‐naive tumors.^[^ ^42^, ^43^, ^44^ ^]^ Consistent with Zheng et al.’s C5_CD8‐GZMK cluster,^[^ ^21^ ^]^ Zheng et al.’s double‐positive T cells^[^ ^45^ ^]^ and Magen et al.’s effector‐like cells,^[^ ^14^ ^]^ PD‐1^+^CD8^+^ Tex‐int cells in *C03_CD8T_DTHD1* showed a “transition” or “bi‐direction” state with an increased expression of both exhaustion (PD‐1, *LAG3*, and *CXCL13*) and activation genes (*GZMK*, *GZMA*), even although they lack *CD39* expression. These results were supported by recent studies on HCC: PD‐1^hi^CD8^+^ T cells in Magen et al.’s study^[^ ^14^ ^]^ and *CD8_C4_LAYN* cluster in Tan et al.’s study^[^ ^46^ ^]^ were specifically enriched in tumor microenvironment and validated as tumor‐reactive CD8^+^ T cells by scTCR‐seq. These subtypes of CD8^+^ T cells also expressed low level of *CD39*, but highly expressed other co‐inhibitory molecules and effector marker. Therefore, PD‐1^+^CD8^+^ Tex‐int cells in *C03_CD8T_DTHD1* were characterized as tumor‐reactive T cells toward tumor‐associated antigens. Moreover, PD‐1^+^CD8^+^ Tex‐int cells expressed T cell recruitment chemokines (*CCL4*, *CCL5*, and *CXCL13*), facilitating the infiltration of effector CD8^+^ T cells into post‐HAIC tumors. According to bulk RNA‐seq cohorts, *C03_CD8T_DTHD1* signature was associated with significantly superior prognosis and higher TLS abundance. Additionally, *C03_CD8T_DTHD1* signature was significantly associated with favorable PFS and OS in patients with advanced HCC receiving HAIC‐based treatment. There are currently no established biomarkers in HAIC treatment for HCC; in this context, PD‐1^+^CD8^+^ Tex‐int cells in *C03_CD8T_DTHD1* showed promise as an effective biomarker for HAIC‐based treatment in HCC.

ST data and multiplex IHC revealed that intermediate‐exhausted Granzyme‐K^+^PD‐1^+^CD8^+^ T cells densely accumulated in TLS niches, particularly in post‐HAIC tumors, suggesting that the direct interplay between PD‐1^+^CD8^+^ Tex‐int cells and TLSs enabled effective T cell immunity. These findings were consistent with literature hypothesis that tumor‐reactive CD8^+^ T cell responses were present in TLS.^[^ ^32^ ^]^ Clinically effective HAIC therapy facilitated the infiltration of Granzyme‐K^+^PD‐1^+^CD8^+^ T cells with functionally antitumor phenotypes, which was another synergetic mechanism with PD‐1 blockade. Moreover, we observed that intermediate‐exhausted Granzyme‐K^+^PD‐1^+^CD8^+^ T cells were predominantly located in the TLS vicinity and gradually decreased with increasing distance to the interface, indicating migration and dissemination of CD8^+^ T cells from TLSs. Accumulating evidence indicates that the dissemination of immune cells within TME is highly interdependent via secreted factors such as chemokines and cytokines.^[^ ^47^ ^]^ However, the mechanisms underlying this process remain to be deciphered. In this study, we identified a special cluster simultaneously expressed signatures of immune cells and fibroblasts in the ST data, suggesting that fibroblasts might participated in the process of immune cell dissemination. Fibroblasts are highly heterogeneous populations in their phenotypes and functions, including promoting cell metastasis, antigen‐presenting, and immune regulatory roles.^[^ ^48^ ^]^ A strong co‐localization of DCN^+^ fibroblasts and cDC2 with active antigen processing and presentation was observed in a recent study, supporting the regulation between CAF subsets and cDC2 through *DCN*.^[^ ^34^ ^]^ In the present study, we observed that DCN^+^ fibroblasts exhibited chemotaxis to CD8^+^ T cell recruitment from in vitro chemotaxis assay. Multiplex IHC also confirmed that Granzyme‐K^+^PD‐1^+^CD8^+^ T cells distributed in linear tracks and in clusters along the DCN^+^ fibroblasts, indicating that DCN^+^ fibroblasts may participate in fibroblastic tracks formation and CD8^+^ T cells dissemination. As DCN^+^ fibroblasts showed significantly higher expression of *CXCL12* compared to DCN^−^ fibroblasts, this dissemination was mainly achieved via the physical interactions and *CXCL12‐CXCR4* axis. Even though fibroblasts were reported to adopt *CXCL12‐CXCR4* axis to restrain T cell response in pancreatic cancer,^[^ ^49^ ^]^ it has also been shown that *CXCL12*‐producing fibroblast mediate lymphocyte migration.^[^ ^50^, ^51^ ^]^ Recently, the co‐occurrence of expression of fibroblast signatures and *CXCL12* was detected by spatial transcriptomics and multiplex IHC in renal cell cancer,^[^ ^52^ ^]^ highlighting the roles of *CXCL12*‐producing fibroblasts for migration and dissemination of plasma cells from TLSs deeper into the tumor cores. These CXCL12^+^ fibroblasts have common properties with the *CXCL12*‐secreting follicular reticular cells,^[^ ^53^ ^]^ or mesenchymal stromal cell‐like fibroblasts^[^ ^54^ ^]^ in the bone marrow, which could promote the dissemination and long‐term survival of plasma cells. Collectively, our study revealed that DCN^+^ fibroblasts might participate in mediating the recruitment, migration, and dissemination of CD8^+^ T cells, and thorough investigation should be carried out in future studies.

There were several limitations to this study. First, due to great heterogeneity of HCC, obtaining tissue from only one puncture point and lacking paired adjacent normal tissue as a control are difficult to represent the TME characteristics of the entire HCC, which unavoidably resulted in certain bias in this integrative analysis. In order to reduce the heterogeneity and improve reliability of our results, we included additional paired pre‐ and post‐HAIC samples for snRNA‐seq, and conducted ST, multiplex IHC, and bulk RNA‐seq for validation cohorts. Consistent results from different cohorts increased the robustness of our findings, but further researches investigating biological mechanisms are necessary. Second, we optimized snRNA‐seq for banked specimens in current study, which had substantial advantages over scRNA‐seq, including reduced dissociation bias, compatibility with frozen tissue, and improved detection of malignant and stromal cells. However, paired TCR sequencing were unable to perform simultaneously for banked frozen samples due to the destruction of cell membrane and loss of membrane receptor information during the single nucleus isolation. Another limitation of this study was the lack of experimental animal models to further explore causality. Transarterial infusion chemotherapy is difficult to conduct on mouse models, due to the substantial differences in anatomy between human and mouse hepatic arterial.

In conclusion, this study demonstrated the cellular and spatial heterogeneity in tumor ecosystem between primary and post‐HAIC tumors, and provided new insights into the cellular underpinnings of HAIC treatment in advanced HCC, which paved the road for designation of optimal HAIC‐immunotherapy combination strategies in future clinical practice. Furthermore, these data also provided a rich resource for the identification of therapeutic targets and candidate biomarkers, which requires further investigation.

## 4 Experimental Section

### Human Subject

A total of 247 patients pathologically diagnosed with HCC at Sun Yat‐sen University Cancer Center were enrolled in this study. Tumor samples from post‐HAIC HCC were referred to as HT group, whereas tumors from paired liver biopsy or primary HCC are referred to as PT group. Patients in the post‐HAIC group generally received 4 cycles of HAIC with FOLFOX regimen before surgical resection, and a small portion of patients received 2 cycles of HAIC treatment due to the superior response and tumor shrinkage.

Primary tumor and adjacent normal tissue, as well as paraffin‐embedded formalin fixed (FFPE) slides of liver biopsy and surgical tumors from HT and PT group were collected. Overall, we collected 16 tumor specimens from 13 patients for snRNA‐seq (discovery cohort: 8 post‐HAIC tumors; 3 paired pre‐treatment liver biopsies, and 5 treatment‐naïve primary tumors). To reduce heterogeneity and improve the reliability of our findings, we included additional paired pre‐treatment and post‐treatment tumors, and conducted snRNA‐seq as Validation cohort 1. Moreover, we collected additional samples to verify the findings of snRNA‐seq. Validation cohort 2: FFPE samples from six treatment‐naive primary or post‐HAIC tumors were collected for ST. Validation cohort 3: FFPE samples from 20 patients for multiplex fluorescence immunohistochemical staining (IHC). The detailed clinical and pathological characteristics were summarized in the Table S1 (Supporting Information). Moreover, we reviewed H&E staining of primary and post‐HAIC HCC (n = 70) to investigate the correlation between TLS density and HAIC treatment. We recruited an additional cohort of HCC with pre‐treatment tumor samples to perform bulk RNA‐seq analysis (n = 135) to investigate the biomarkers for HAIC‐based treatment in patients with HCC. This study was approved by the Institutional Review Board of Sun Yat‐sen University Cancer Center (No. B2024‐019‐02) and performed in accordance with the Declaration of Helsinki.

### Single‐Nucleus RNA‐Seq (snRNA‐Seq)

*Sample Processing and Library Construction*: Single nucleus isolation from frozen tumor samples were conducted according to the standard protocols, and nucleus suspension with an optimal concentration was obtained after passing through a 40 µm cell filter. Approximately 14 000 nuclei per sample were then loaded on a Chromium controller. Single‐nucleus RNA‐seq libraries were prepared using the Chromium Next GEM Single Cell 30 Kit from 10X Genomics, following the manufacturer's instructions. Single nuclei were partitioned into droplets with gel beads in the Chromium Controller to form emulsions, after which the nuclear lysis, barcoded reverse transcription of mRNA, cDNA amplification, and the other library construction steps were performed according to manufacturer's instructions. Single‐nucleus RNA‐seq libraries were sequenced on the Illumina NovaSeq 6000 sequencing system (paired‐end multiplexing run, 150 bp) by LC‐Bio Technology Co., Ltd, (Hangzhou, China).

### snRNA‐Seq Data Pre‐Processing

The CellRanger version 7.0.0 (10X Genomics) was used to align reads to the human reference genome (GRCh38), and the raw count matrix for each sample was obtained from the CellRanger unique molecular identifier (UMI) matrix output. Quality control and integration were performed by several steps using Seurat^[^ ^55^ ^]^ v5 package. First, genes covered by less than 3 cells were filtered out. Then, all cells expressing < 200 or > 7500 genes or expressing high mitochondrial gene (>5%) were removed to filter out the most of barcodes associated with empty partitions or doublet cells. Finally, DoubletFinder^[^ ^56^ ^]^ package was applied to identify and remove doublets. This resulted in a total of 126809 high‐quality single nucleus transcriptome for the downstream analysis. To integrate and embed single cells from different samples into a shared low‐dimension space, we used integrated analyses by the function “‘IntegrateData” to perform batch effect correction and normalization.

### Dimension Reduction, Unsupervised Clustering, and Identification of Broad Cell Type

Dimension reduction and unsupervised clustering were applied to the single‐cell data following the workflow in Seurat v5. In brief, the top 2000 highly variable genes were identified and 20 principal component (PCs) were applied to reduce dimensionality. We used the function “FindClusters” with resolution 1.2 to perform the first‐round cluster and annotated each cluster by known markers (Hepatocyte: *HNF4A*, *ALDH1A1*, *GPC3*; Fibroblast: *ACTA2*, *COL1A1*, *COL1A2*, *DCN*; Endothelial cells: *CD34*, *VWF*, *CDH5*; T/NK cells: *CD2*, *IL7R*, *CD247*; B/Plasma cells: *MS4A1*, *BANK1*, *MZB1*; Myeloid cells: *CD163*, *CD86*, *FCN1*, *FLT3*). For visualization, the dimensionality of each dataset was further reduced using Uniform Manifold Approximation and Projection (UMAP) with Seurat function “RunUMAP”.

### Re‐Clustering of Broad Cell Types

The initial labeling resulted in the identification of hepatocyte, endothelial, fibroblast, myeloid, T/NK, and B/plasma cell populations. We split the Seurat objects into subsets of the broad cell types, and reran the Seurat pipeline, including integration with different methods (canonical correlation analyses, CCA^[^ ^57^ ^]^; Harmony^[^ ^58^ ^]^), scaling, PCA, UMAP dimension reduction, clustering and DGE analyses on each broad cell type. To annotate subpopulations within each major cell cluster, we determined the cell cluster identities by screening top‐ranked DEGs and previously reported biological related genes.

Using T cell markers (*CD2*, *IL7R*, *CD247*), a total of 12323 T cells were identified to reveal 13 clusters, which were annotated according to top‐ranked DEGs and published signatures, including CD4^+^, CD8^+^ T, and NK cells. Using myeloid cell markers (*CD163*, *CD86*), 7876 myeloid cells were identified to reveal 12 clusters, including dendritic cell, macrophage, and monocyte‐like subsets. Using fibroblast markers (*ACTA2*, *COL1A1*), we identified a total of 4376 fibroblast cells, which were classified into myofibroblast or annotated according to marker genes. Using B cell markers (*MS4A1*, *BANK1*, *MZB1*), we identified five B cell phenotypes, which were annotated as naïve, memory B, and plasma cells. Using hepatocytes markers (*HNF4A*, *ALDH1A1*, *GPC3*), a total of 87319 cells were identified. To identify malignant cells, CopyKAT^[^ ^59^ ^]^ algorithm was utilized to estimate the copy number variation (CNVs). The fibroblast and myeloid cells were used as normal reference, and the parameters were default. As a result, we identified 61779 aneuploid cells, which were then annotated as malignant hepatocytes and included for downstream analyses.

### Differential Expression and Gene Ontology Enrichment Analysis

To characterize and identify the marker genes for each cluster, differential expression genes (DEGs) were performed using the function “FindAllMarkers” and “FindMarkers” to identify positive (overexpressed) markers in each population. Genes with adjusted *P* value < 0.05 by Wilcoxon rank‐sum test were defined as cluster‐specific signature genes. Gene Ontology (GO) and pathway enrichment analyses of DEGs were performed using the “clusterProfiler”^[^ ^60^ ^]^ package. GO terms with adjusted *P* values < 0.05, using the BH procedure, were considered significant.

### Cell Developmental Trajectory

The cell lineage trajectory of CD8^+^ T cells was inferred by using Monocle 2.^[^ ^61^ ^]^ For Monocle 2, the new CellDataSet objects were built from cluster‐annotated Seurat objects using the “newCellDataSet” function. Then, the “differentialGeneTest” function was used to derive DEG from each cluster, and genes with q‐value < 0.001 were used to order the cells in pseudotime. Dimension reduction was performed using the DDRTree algorithm and then cells were ordered along the trajectory.

### Cell‐to‐Cell Communication of snRNA‐Seq Data

The number and strength of cell‐to‐cell interactions between malignant, immune, and stromal cells were systematically inferred and visualized by R toolkit CellChat^[^ ^62^ ^]^ using the standard framework. CellChat infer biologically significant cell‐cell communication from scRNA‐seq data according to the curated ligand‐receptor interaction database. We compared the outgoing and incoming interaction strength of each pair of cell types to identify significant changes in sending or receiving signals between HT and PT groups, and used the interaction strength to qualify the HAIC treatment effect.

### Cell Subtype Deconvolution from Bulk RNA‐Seq

To assess the function of malignant cell subtypes in larger compendiums of samples, we assessed their composition in bulk RNA‐seq data from The Cancer Genome Atlas‐liver hepatocellular carcinoma^[^ ^63^ ^]^ (TCGA‐LIHC) and Fudan‐HCC^[^ ^36^ ^]^ cohort. We used a deconvolution algorithm Bayesprism^[^ ^64^ ^]^ with a standard framework to simulate the cell‐type‐specific gene expression profiles for predicting the abundance of each malignant cell subtypes quantified by snRNA‐seq. As a result, the abundance of each malignant cell subtype in all HCC patients was obtained. To identify the potential HCC subtypes, the HCC patients were partitioned using the k‐means clustering method based on the absolute or normalized abundance of malignant cell subtypes inferred from bulk RNA‐seq.

### Definition of Cell Scores and Gene Signatures

For computation of signature scores, average expression of the signature genes was counted based on the normalized matrix generating by Seurat Package. Gene signatures used in the present study were collected from previous cancer related studies and were provided in Table S3 (Supporting Information). To define the potential phenotype in malignant hepatocytes in PT and HT group, we calculated the signature score of 50 hallmark gene sets (derived from MSigDB) as previously described.^[^ ^65^ ^]^ To identify statistically significant changes of gene signatures between PT and HT group, “FindAllMarkers” function was used.

To confirm the connections of distinct immune cell clusters identified by the single‐cell analysis, the signature genes of immune cell clusters were utilized and calculated the expression scores in bulk RNA‐seq datasets of TCGA‐LIHC and Fudan HCC. The cell‐type specific gene signatures of immune cell clusters were defined according to the top DEGs and literature‐based knowledge. Due to the different transcriptomic distribution between bulk RNA‐seq and snRNA‐seq, ssGSEA was applied using standard settings to calculated signatures derived from bulk RNA‐seq.

### Spatial Transcriptomics Sequencing (Visium CytAssist, 10X Genomics)

FFPE samples passing the RNA quality control (DV200 > 30%) were used for spatial transcriptomics construction and sequencing according to the recommended protocols. After deparaffinization, H&E staining, imaging and decrosslinking, probe hybridization was performed on selected tissues based on Visium CytAssist Spatial Gene Expression Reagent Kits. Probe‐based library construction and sequencing were performed on the Illumina platform through the usage of spatially barcoded oligonucleotides with default protocols by 10X genomics platform, performed by CapitalBio Technology, Beijing.

### Spatial Transcriptomics Data Analysis

Raw sequencing data of spatial transcriptomics and manually aligned histology images were analyzed with Space Ranger (version 1.3.0) to detect tissue, align reads, generate feature‐spot matrices. An average of 5789 median genes per spot was measured, with median genes per spot ranging from 3812 to 8099. The generated feature‐spot matrices were analyzed with the Seurat package. Spatial transcriptome data were qualitatively controlled using parameters including total spots, median UMI/spot, median genes/spot, and median mitochondrial genes/spot. Spatial spots featuring > 30% of mitochondrial genes and < 300 genes were filtered out, as they identified as necrotic or damaged tissue areas that were validated by an experienced pathologist. Genes with counts in less than 5 spatial spots were also discarded. After quality control, normalization, highly variable genes, and scaling data across spots were performed with the “SCTransform” function. Dimensionality reduction and clustering were performed with the principal component analysis (PCA) with the first 20 PCs. Additionally, to compare the clusters at gene level, differentially expressed genes were identified of all or selected clusters using fold change analysis and Wilcoxon rank sum test. Based on the unbiased clustering and spot features, spots of tumor tissues were classified as hepatocytes with higher expression of *ALB* and *ADH1B*, fibroblast with high expression of *COL1A1, DCN*, and *VCAN*, myofibroblasts with high expression of *MYH11* and *TAGLN*, immune cells with higher expression of *PTPRC*, *CD3D*, *IL7R*, *MS4A1*, *IGHG1* and *MZB1*, endothelial with *VWF* and *CD34*, cholangiocytes with high expression of *FXYD2* and *MUC1*, and malignant hepatocytes with higher expression of *G6PC*, *GPC3* and *AFP*.

To better exhibition spatial expression of features, the spots were enhanced using the “spatialEnhance” function of BayesSpace,^[^ ^66^ ^]^ the expression genes were enhanced with “enhanceFeatures” function. Signature score derived from snRNA‐seq data was added to “metadata” of ST dataset using “AddModulScore” function with default parameters in Seurat. Spatial feature expression plots were generated with the “SpatialFeaturePlot” function in Seurat package.

### Generation of Stable Cell Lines and Cell Culture

The mouse fibroblast cell line L929 was purchased from the Cell Bank of the Chinese Academy of Sciences (Shanghai, China). Cell lines were maintained in a special culture medium supplemented with 10% FBS, penicillin (100 mg mL^−1^) and streptomycin (100 u mL^−1^) and were cultured in a humidified atmosphere of 5% carbon dioxide at 37 °C. To obtain a stable *Dcn* overexpression mouse fibroblast cell line (L929), lentiviral particles were generated by cotransfecting OE‐Dcn plasmid with packaging plasmid mix using TurboFect transfection reagent in HEK‐293T cells and harvested 48 h later. L929 cells were then infected with a proper amount of virus in the presence of polybrene at a final concentration of 10 µg mL^−1^. After 48 h of infection, L929 cells were then selected in the presence of 6 µg mL^−1^ puromycin to obtain a stable cell line for follow‐up experiments. Quantitative reverse transcription real‐time polymerase chain reaction (qRT‐PCR) was performed to determine the overexpression efficiency, respectively.

### ELISA

Concentrations of Decorin from the supernatant of the in vitro culture system were detected using ELISA kits according to the manufacturer's instructions.

### Chemotaxis Assay

Transmigration of mouse primary CD8^+^ T cells was assessed in 6.5‐mm diameter 24‐Transwell chemotaxis chambers. Briefly, equal numbers of OE‐Dcn, OE‐vector, or WT mouse fibroblast cell line L929 were added to the lower chamber of each transwell for 24 h. Lower chambers were filled with culture medium, in the presence or absence of different doses of recombinant mouse *Cxcl12* or AMD3100. The mouse CD8^+^ T cells were sorted and activated by the anti‐mouse CD3/CD28 antibody for 24 h. Then, CD8^+^ T cells were added to the upper layer of the transwell 24 h later. After 12‐h incubation, medium in the lower chamber was collected, and the suspended cells that migrated were manually counted under a light microscope.

### Histology Evaluation of Human Tumor Tissue Sections

Paraffin‐embedded tumor samples from treated naive primary and post‐HAIC patients were cut in 4‐µm consecutive sections and processed for H&E staining, IHC of tissue microarrays (TMA), and multicolor immunohistochemistry.

### Tertiary Lymphoid Structure (TLS) Calling from H&E Staining

TLS were identified and quantified by H&E staining of FFPE samples. TLSs can be classified as lymphoid aggregates (Agg) and lymphoid follicles (Fol); Fol can be further subdivided into Fol‐I (without germinal centers) or Fol‐II (with germinal centers). TLS abundance in the tumor region can be graded into 4 categories as described in previous study^[^ ^67^ ^]^: (a) score 0 indicates no TLS in the tumor region; (b) score 1 represent the tumor region with 1‐2 TLSs; (c) score 2 indicates at least 3 TLSs in the tumor region but does not meet the criteria of score 3; (d) score 3 represents a large number of TLSs distributed throughout the tumor region that converge with each other. The TLS score was assessed independently by two experienced pathologists who were blinded to the clinical data.

### Multiplex Fluorescence Immunohistochemistry

Tumor samples from treatment naive primary and post‐HAIC patients with HCC were subjected to multiplex staining and multispectral imaging using the PANO 7‐plex IHC Kit (Panovue) based on the manufacturer's protocol. In brief, FFPE tissue sections were dewaxed, rehydrated and subjected to high‐temperature antigen retrieval. And different primary antibodies were sequentially applied, followed by HRP‐conjugated secondary antibody incubation and tyramide signal amplification working solution. Next, slides were subjected to microwave heat‐treated antigen retrieval. Finally, nuclei were stained with DAPI (Sigma‐Aldrich) after all the primary antigens had been labeled. Multiplex IHC were performed with the same protocols but different primary antibodies for two panels containing antibodies against: Panel 1: CD8A (Cell Signaling Technology), CD4 (ZSGB‐BIO), CD20 (Abcam), CD21 (Abcam), GZMK (Abcam), PD‐1 (Cell Signaling Technology); Panel 2: CD8A (Cell Signaling Technology), GZMK (Abcam), PD1 (Cell Signaling Technology), SMA (ZSGB‐BIO), and DCN (Abcam). Whole scanned images were obtained with the Akoya Vectra Polaris slide scanner at 200× magnification. The percentage of a cell subtype was evaluated using the co‐localized signals for DAPI and the corresponding cell subtype markers. Quantification of individual and co‐expressing markers in the multicolor images was performed with HALO. The presence of TLS was determined by immunofluorescence staining, with the aggregates of CD4/CD8^+^ T and CD20^+^ B cells.

### Statistical Analysis

Bulk RNA‐seq and clinical data of TCGA‐LIHC and Fudan HCC cohorts were used to evaluate the prognostic performance of specific immune cell clusters. For all survival analyses of bulk RNA‐seq samples stratified by snRNA‐seq cell types, signature scores of cell cluster were calculated by ssGSEA, and the samples were grouped into high and low expression groups by the median value. Survival curves were performed using the Kaplan‐Meier method with the Survival package, and visualized using the “ggsurvplot” function of the survminer package. The significance was evaluated by the log‐rank test. Comparisons of gene signature or cell distribution between two groups were performed using unpaired Wilcoxon rank‐sum test or Student t test. *P*‐value < 0.05 was considered statistically significant. SPSS, R, and GraphPad‐Prism were used for statistical analysis.

### Ethics Statements

This study was approved by the Institutional Review Board of Sun Yat‐sen University Cancer Center and performed in accordance with the Declaration of Helsinki. Written informed consent was obtained from all patients.

## Conflict of Interest

The authors declare no conflict of interest.

## Author Contributions

Y.X.H., Z.F.D., and Z.C.L. contributed equally to this work. M.S., A.K., and Y.X.H. conceptualized and designed this study. Y.X.H., Z.F.D, Z.C.L, D.S.W., L.C.H., M.K.H., H.F.L., and A.K. performed the methodology. Y.X.H., Z.F.D., Z.C.W., and H.Y.O.Y. performed bioinformatic data analyses or interpreted the results. Y.X.H., Z.F.D, Z.C.L, W.C.W., H.F.L., and A.K. provided valuable discussions. Y.X.H. wrote the manuscript. M.S. and A.K. performed the review and editing. L.Z.C., A.K., and M.S. acquired the funding. M.S. supervised the study.

## Supporting information



Supporting Information

Supporting Information

## Data Availability

The data that support the findings of this study are available from the corresponding author upon reasonable request.
