# Supplementary material for: Single‐Nucleus and Spatial Transcriptome Profiling Delineates the Multicellular Ecosystem in Hepatocellular Carcinoma After Hepatic Arterial Infusion Chemotherapy
Source: Adv Sci (Weinh). 2024 Dec 16;12(5):2405749. doi: 10.1002/advs.202405749 (PMC11791974; doi:10.1002/advs.202405749)
Supplement: Supplementary file 1 — Supporting Information [file ADVS-12-2405749-s002.docx]

**Supplementary Materials for**

Single-Nucleus and Spatial Transcriptome Profiling Delineates the Multicellular Ecosystem in Hepatocellular Carcinoma after Hepatic Arterial Infusion Chemotherapy

YeXing Huang*; ZeFeng Du*; ZhiCheng Lai*; DongSheng Wen; LiChang Huang; MinKe He; ZiChao Wu; HuiFang Li; HanYue OuYang; WenChao Wu; Anna Kan^#^; Ming Shi^#^

Correspondence to: [shiming@mail.sysu.edu.cn](mailto:shiming@mail.sysu.edu.cn); [annakan@sysucc.org.cn](mailto:annakan@sysucc.org.cn)

**This PDF file includes:**

Supplementary Figures. S1 to S8

**
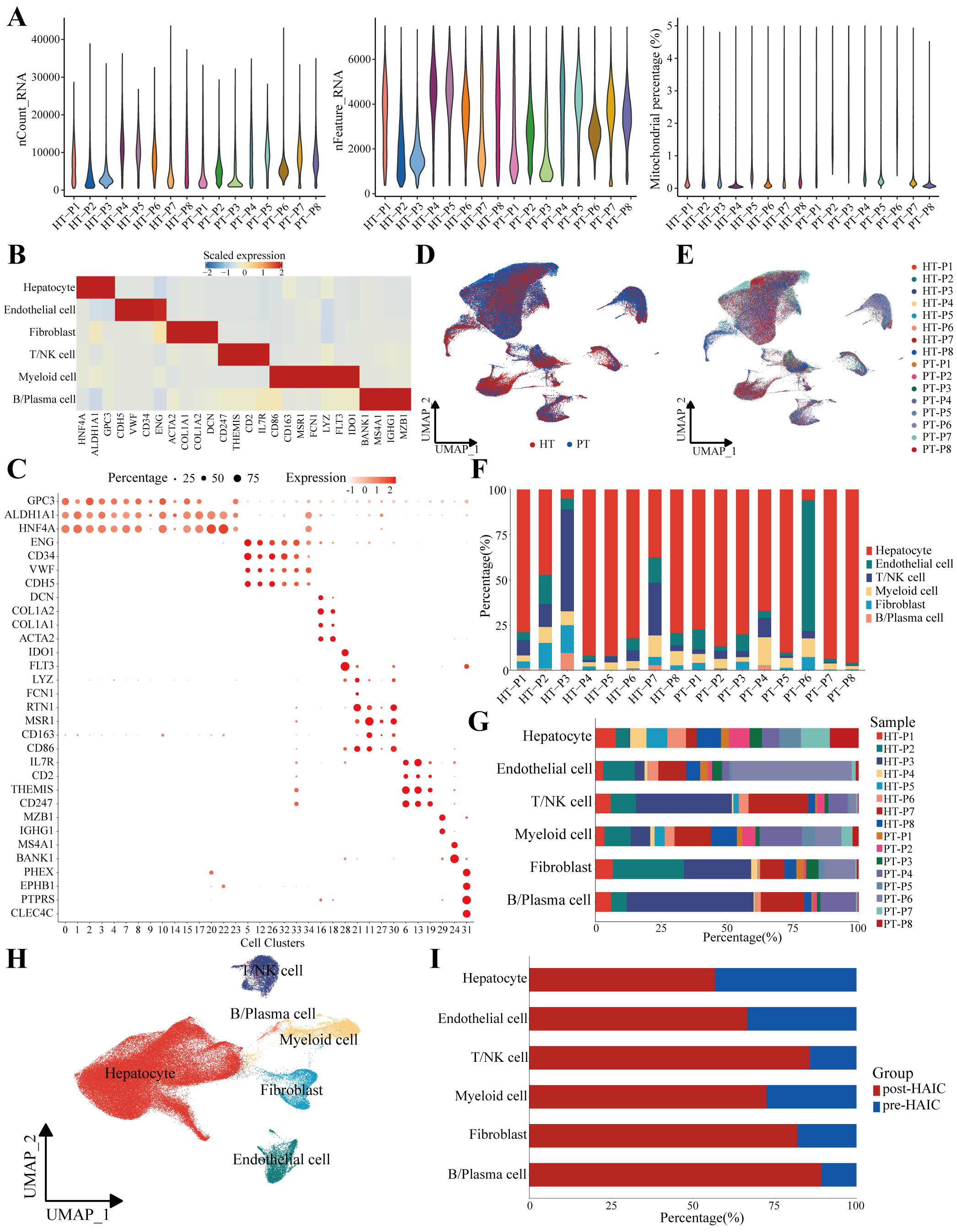
Figure S1: snRNA-seq profiling of the tumor ecosystem in** **primary and** **post-HAIC HCC, related to Figure 1.**

(A) Violin plots showing the quality control features, including the number of UMI count (left panel), gene feature (middle panel), and percentage of mitochondrial genes (right panel) of each patient.

(B) The heatmap showing the expression of well-known marker genes in the major cell types.

(C) Dotplot showing marker genes across 35 clusters from 126,809 high-quality cells.

(D, E) The UMAP plots showing cell origins by colors: HT or PT origin (D), and patient origin (E).

(F) Bar plots indicating the proportions of major cell types for each patient.

(G) Bar plots indicating the fractions of major cell types originating from each patient. (H) The UMAP plot showing the major cell types in the validation cohort 1.

(I) Bar plots indicating the fractions of major cell types originating from the pre- and post-HAIC groups in the validation cohort 1.

**
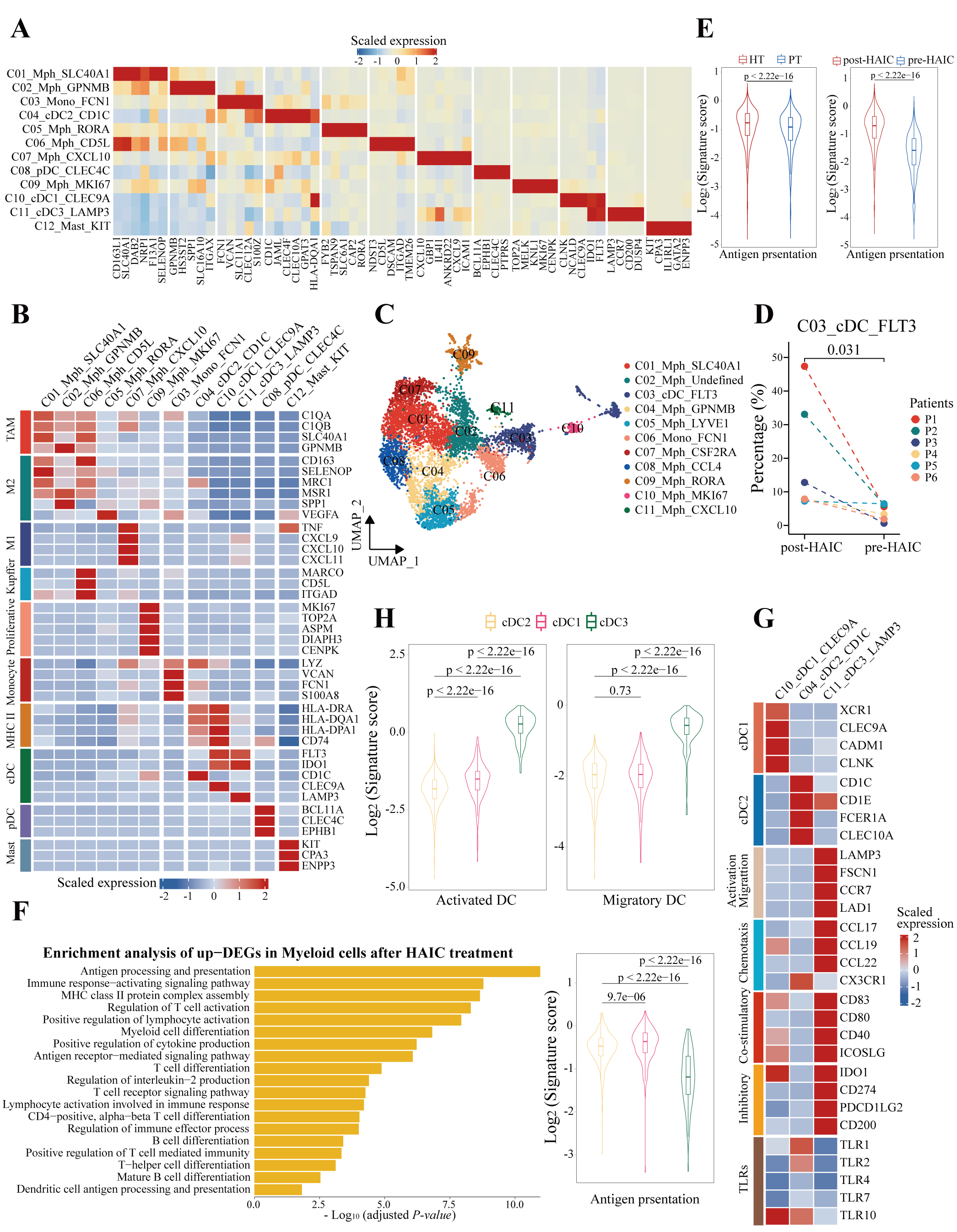
Figure S2: Characterization of the heterogeneity of intratumoral myeloid cells, related to Figure 1.**

(A) Heatmap showing the expression of marker genes in the myeloid cell subtypes.

(B) Heatmap indicating the expression of selected gene sets in the myeloid cell subtypes.

(C) The UMAP plot showing the subtypes of myeloid cells in the validation cohort 1.

(D) Comparison of percentage of cluster *C03_cDC_FLT3* in myeloid subtypes between paired pre- and post-HAIC samples in the validation cohort 1. (each group, n = 6).

(E) The violin plot showing the signature scores of antigen presentation in myeloid cells from HT and PT samples (left panel), or from paired pre- and post-HAIC samples (right panel), and compared with a two-sided Wilcoxon test.

(F) Bar chart showing GO pathway enrichment analysis of up-regulated genes between paired pre- and post-HAIC samples among myeloid cells in the validation cohort 1.

(G) Heatmap indicating the expression of selected gene sets in dendritic cell subtypes.

(H) Violin plots showing the expression scores of activated DC, migratory DC, antigen presentation signatures from different dendritic cell subtypes, and compared with a two-sided Wilcoxon test.

**
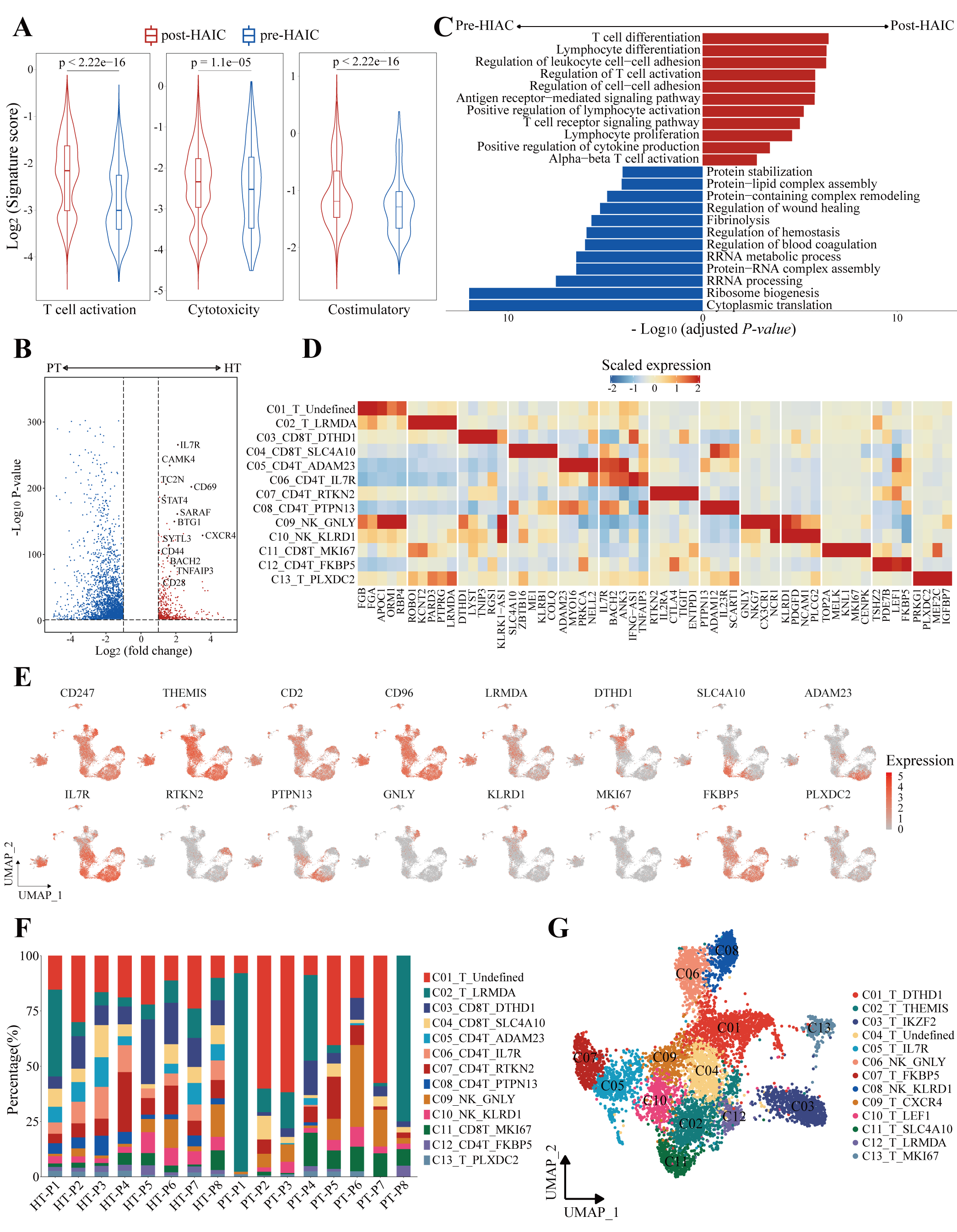
Figure S3: Distinct T cell compositions and states between primary and post-HAIC HCC, related to Figure 2.**

(A) Violin plots showing the expression scores of T cell activation, cytotoxicity, and co-stimulatory signatures in T/NK cells from paired pre- and post-HAIC samples in the validation cohort 1, and compared with a two-sided Wilcoxon test.

(B) Volcano plot showing the differentially expressed genes between HT vs. PT groups among T/NK clusters.

(C) Bar chart showing GO pathway enrichment analysis of differential expressed genes between paired pre- and post-HAIC samples among T/NK cells in validation cohort 1.

(D) Heatmap showing the expression of marker genes in the T/NK subtypes.

(E) UMAP plots showing the expression of marker genes from the T/NK subtypes.

(F) Bar plots indicating the proportions of T/NK cell subtypes for each patient.

(G) UMAP plot showing the subtypes of T/NK cells in the validation cohort 1.

**
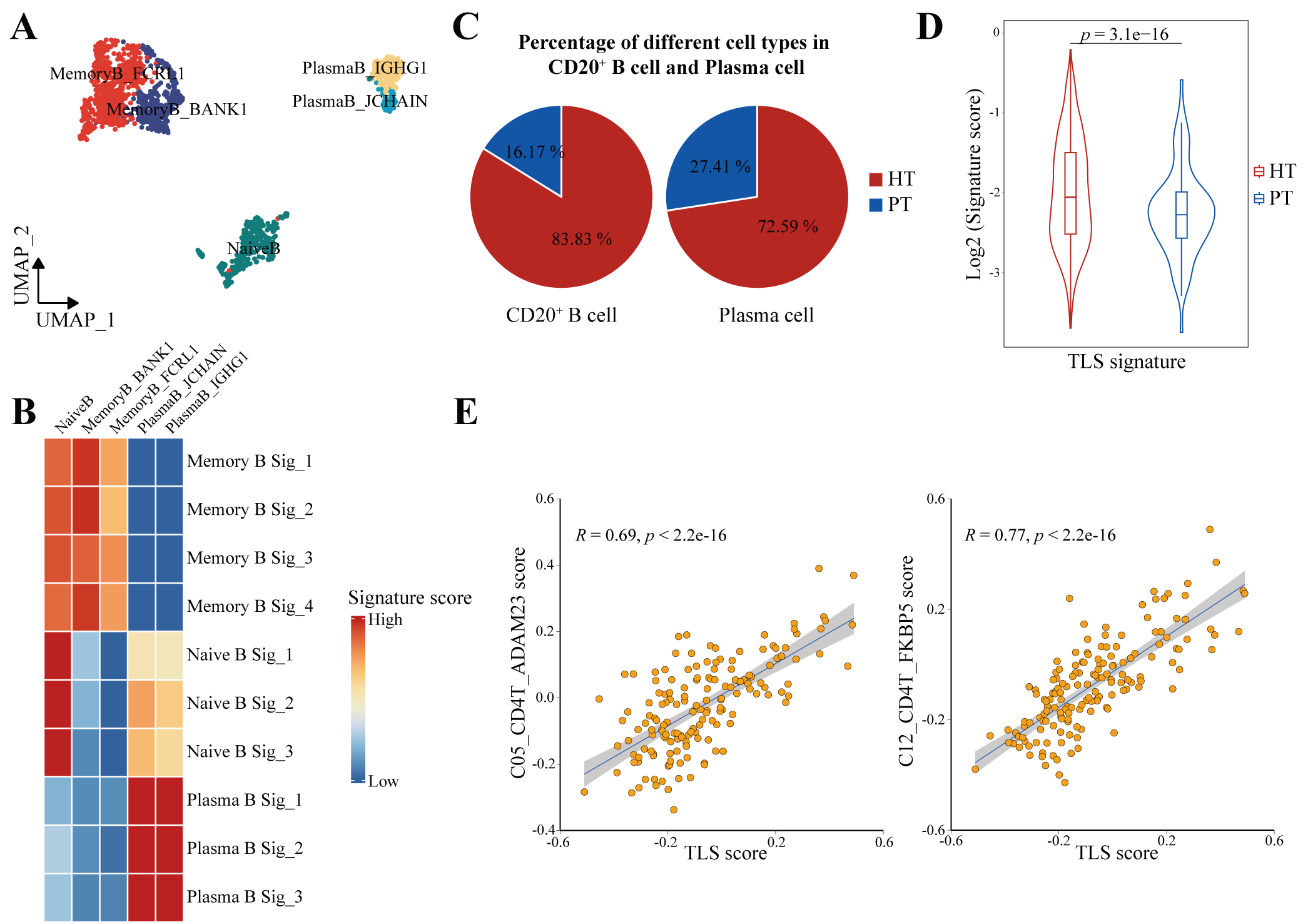
Figure S4: Characterization of the heterogeneity of intratumoral B/Plasma cells, related to Figure 3.**

(A) UMAP plot showing the subtypes of B/Plasma cells.

(B) Heatmap showing the enrichment score of selected gene signatures across B/Plasma cell subtypes.

(C) Pie charts showing percentages of HT and PT samples in each B/Plasma subtype.

(D) Violin plot showing the expression scores of TLS signature in B cells from HT and PT samples, and compared with a two-sided Wilcoxon test.

(E) Scatterplots showing the correlation of T cells in cluster *C05_CD4T_ADAM23* and *C12_CD4T_FKBP5* with TLS in Fudan-HCC cohort according to their signature genes expression.

**
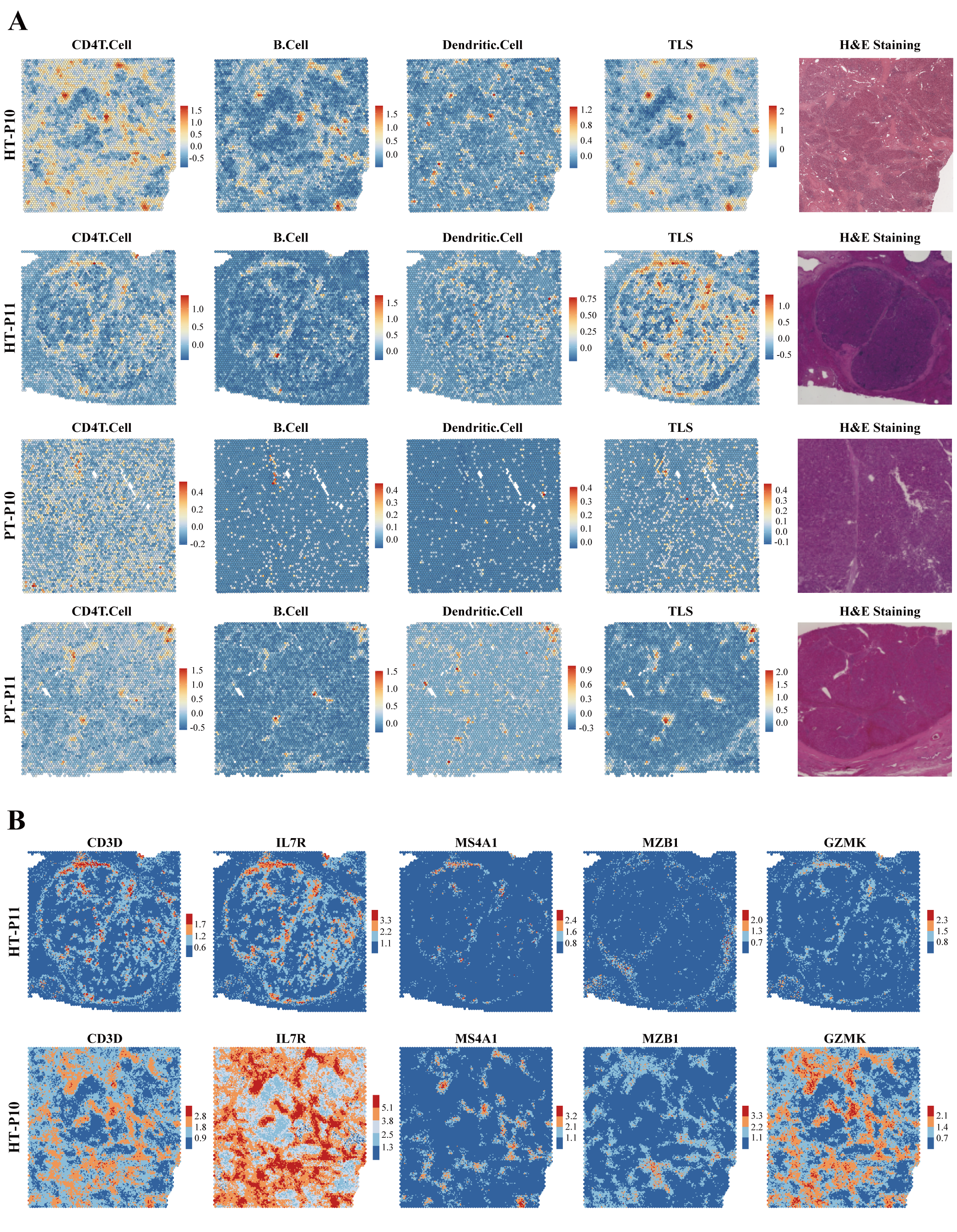
Figure S5: Spatial co-localization of CD4^+^ T, cDCs, and CD20^+^ B cells, related to Figure 4.**

(A) Spatial feature plots showing the signature scores of selected immune subtypes in spatial sections of the other four patients. Pathologist’s annotations of the TLS areas on H&E staining used for the spatial transcriptomics assay were shown in right panel.

(B) Spatial feature plots showing the enhanced expression of markers for T cells (*CD3D*, *IL7R*), B cells (*MS4A1*, *MZB1*), and cytotoxicity (*GZMK*) in other two patients of HT group, calculated by BayesSpace algorithm.

**
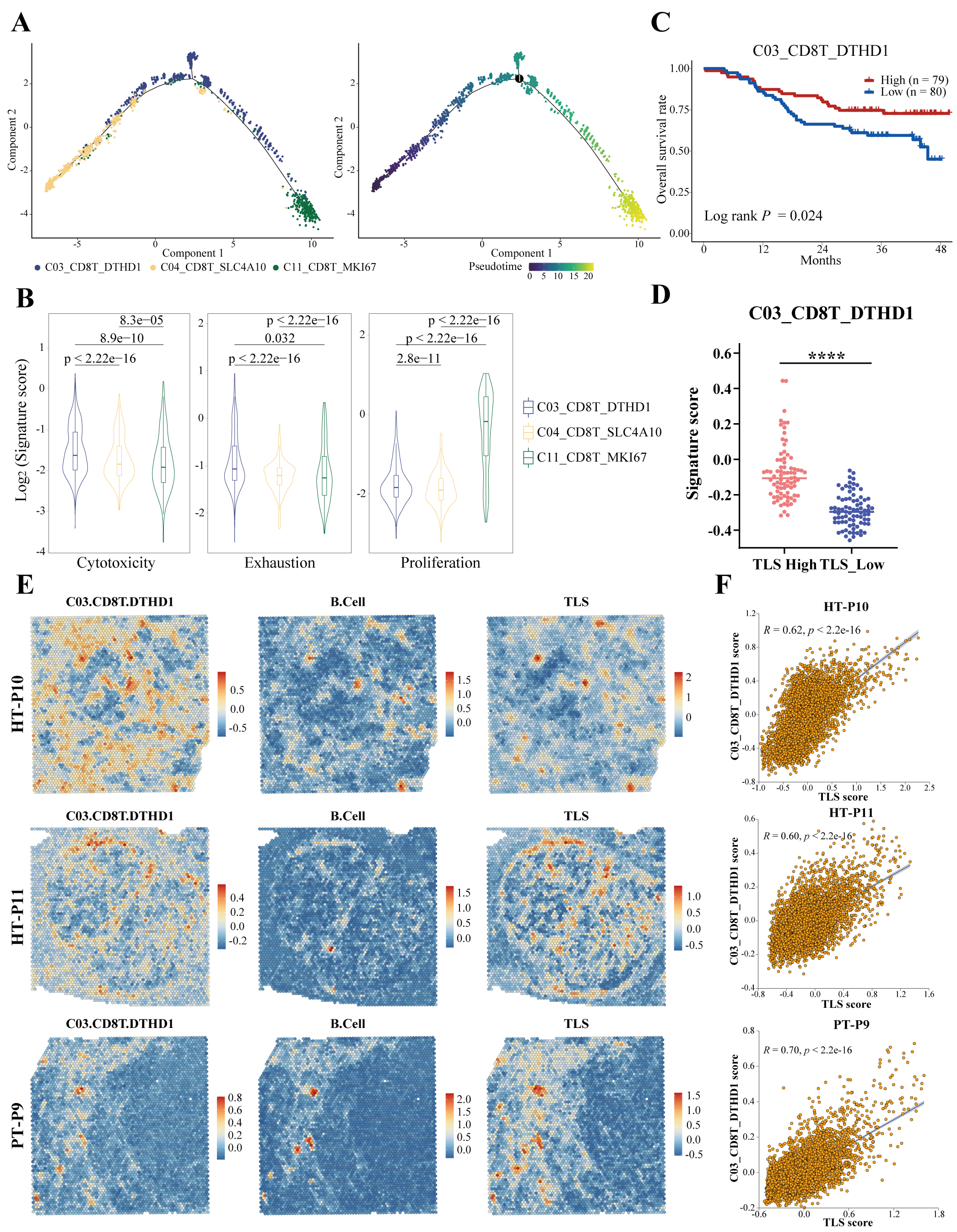
Figure S6: Intermediate-exhausted CD8^+^ T subtype increases following HAIC and exhibits functionally antitumor phenotype, related to Figure 5.**

(A) Pseudotime-ordered analysis of CD8^+^ T cell clusters from all samples.

(B) Violin plots showing expression scores of cytotoxicity, exhaustion, and proliferation signatures from different CD8^+^ T subtypes, and compared with a two-sided Wilcoxon test.

(C) Kaplan-Meier overall survival curves of Fudan-HCC cohorts, grouped by the gene signature expression of cluster *C03_CD8T_DTHD1*.

(D) The scatterplot showing the comparison of *C03_CD8T_DTHD1* signature scores between the TLS-high group and the TLS-low group based on the Fudan-HCC cohort. * *p* < 0.05, ** *p* < 0.01, *** *p* < 0.001, **** *p* < 0.0001.

(E) Spatial feature plots showing the signature scores of selected immune subtypes in spatial sections of other three representative patients.

(F) Scatterplot showing the correlation analysis of signature score *C03_CD8T_DTHD1* and TLS in spatial spots of other three representative patients.

**
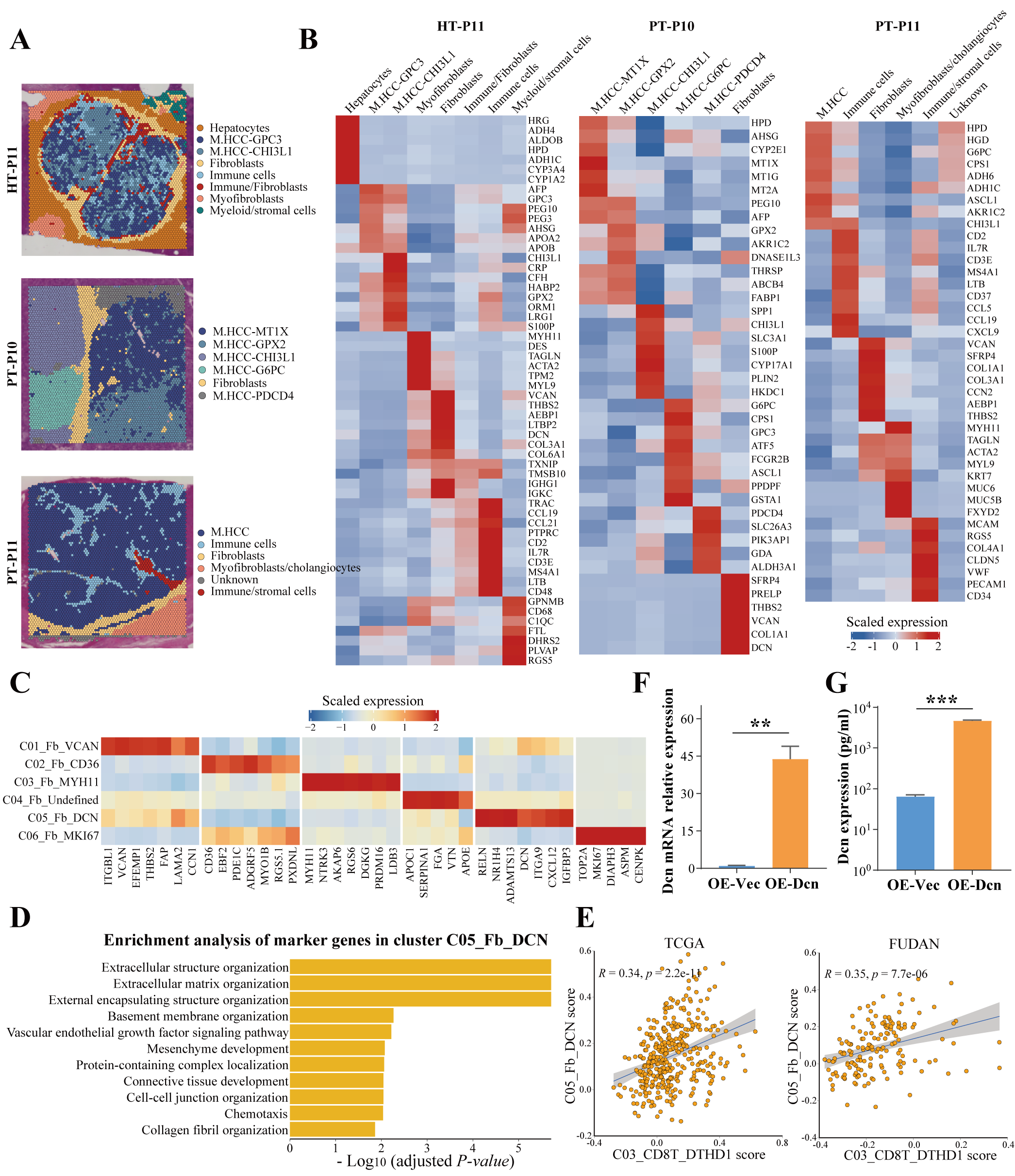
Figure S7: Intermediate-exhausted CD8^+^ T** **subtype accumulates in TLSs and disseminates along** **fibroblastic tracks****, related to Figure 6-7.**

(A) Unbiased clustering of ST spots and definition of cell types of each cluster in tumor sections of the other three representative patients.

(B) Heatmap showing the average expression of known markers in indicated clusters.

(C) Heatmap showing the expression of marker genes in the fibroblast subtypes.

(D) Bar chart showing GO pathway analyses of marker genes in cluster *C05_Fb_DCN*.

(E) Scatterplots showing the correlation of *C03_CD8T_DTHD1* and *C05_Fb_DCN* in TCGA-LIHC and Fudan-HCC cohorts based on their signature genes expression.

(F) Relative mRNA expression level of Dcn measured by RT-qPCR in OE-Dcn vs. OE-Vec L929 cell line. (each group, n = 3).

(G) ELISA assay of *Dcn* secretion in an in vitro cultured OE-Dcn vs. OE-Vec L929 cell line. (each group, n = 12), * *p* < 0.05, ** *p* < 0.01, *** *p* < 0.001, **** *p* < 0.0001.

**
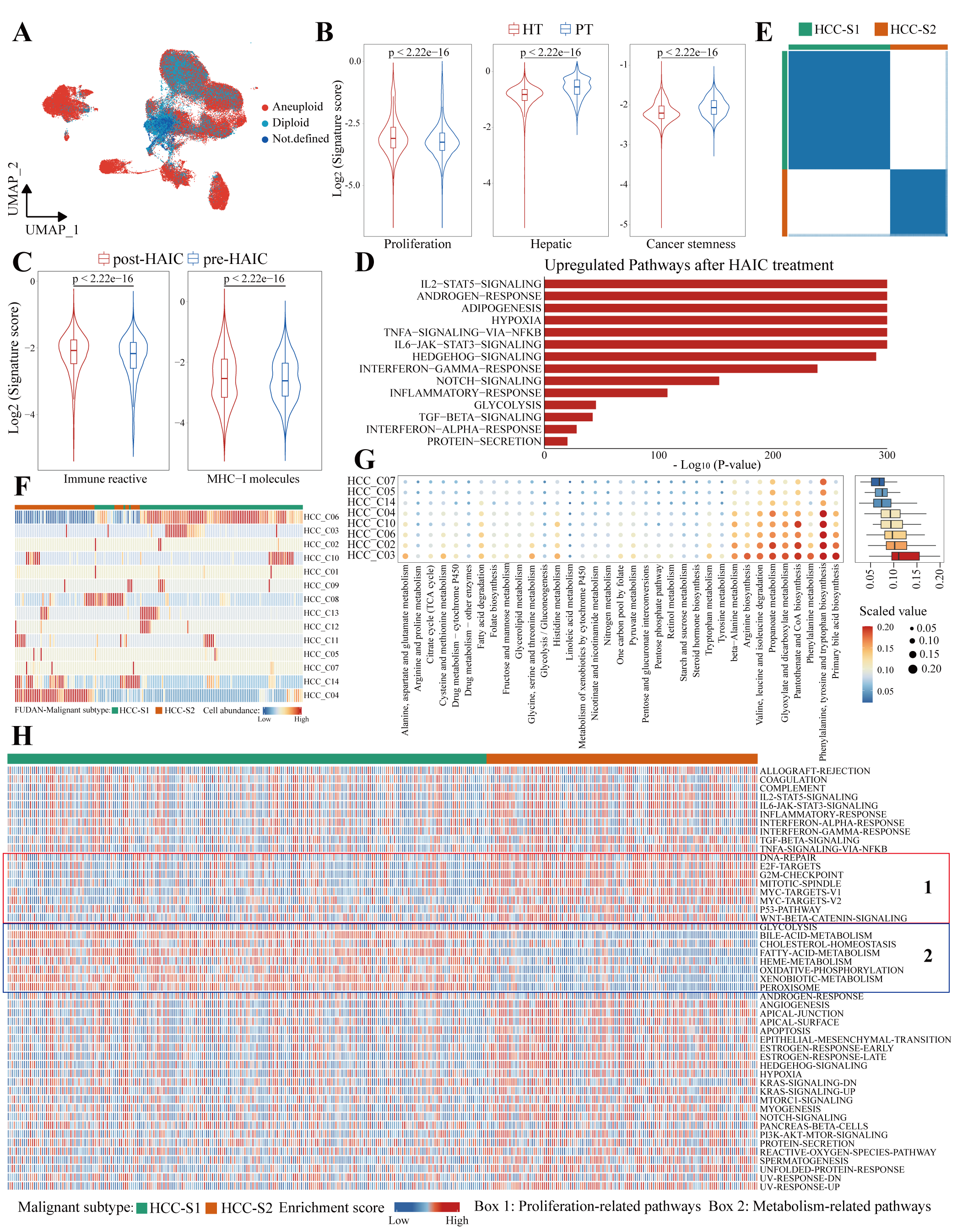
Figure S8: Characterization of the transcriptomic heterogeneity of intratumoral** **malignant hepatocytes, related to Figure 8.**

(A) UMAP plot showing the CopyKAT analyses of hepatocytes. Cells from different CopyKAT results are marked by colors.

(B) Violin plots showing the signature scores of proliferation, hepatic, cancer stemness in malignant hepatocytes from the HT and PT samples, and compared with a two-sided Wilcoxon test.

(C) Violin plots showing the signature scores of immune reactive and MHC-I molecules in malignant cells from paired pre- and post-HAIC samples in the validation cohort 1, and compared with a two-sided Wilcoxon test.

(D) Bar charts showing the enrichment of specific pathways, according to the hallmark gene sets in malignant cells from paired pre- and post-HAIC samples in the validation cohort 1.

(E) Heatmap showing two malignant subtypes of HCC patients identified by consensus clustering using the estimated abundance of malignant cell subtypes.

(F) Heatmap showing two malignant subtypes of HCC patients, with distinct abundance of malignant cell subtypes inferred from Fudan-HCC cohort.

(G) Dot plots showing the metabolic activity analysis of malignant hepatocyte subtypes. The circle size and color both represent the scaled metabolic score.

(H) Heatmap showing the hallmark landscape of two malignant subtypes of HCC patients in TCGA-LIHC cohort.
